# Jacobi-Ritz formulation for modal analysis of thick, anisotropic and non-uniform electric motor stator assemblies considering axisymmetric vibration modes

**DOI:** 10.1007/s11012-025-02071-6

**Published:** 2025-12-24

**Authors:** Panagiotis Andreou, Amal Z. Hajjaj, Mahdi Mohammadpour, Stephanos Theodossiades

**Affiliations:** School of Mechanical, Electrical and Manufacturing Engineering, Loughborough University, Wolfson Building, Ashby Rd, Loughborough, Leicestershire, LE11 3TU UK

**Keywords:** Jacobi-Ritz, Modal analysis, Stator, Analytical, Experimental

## Abstract

This study presents a novel and efficient modal analysis framework for thick cylindrical structures with complex geometry and material variability, subject to arbitrary boundary conditions. The methodology is applied to an electric motor (e-motor) stator assembly modelled as a thick cylindrical shell incorporating stator teeth, windings, and housing or cooling jacket effects. The model accommodates both continuous and piecewise variations in material properties and thickness. Based on First-Order Shear Deformation Shell Theory (FSDST), it accounts for shear deformation, rotary inertia, and trapezoidal stress distributions, enabling accurate prediction of axial, circumferential, torsional, and bending vibration modes. A segmentation approach is used along the axial direction, with artificial massless springs enforcing continuity and permitting general boundary conditions. Displacement fields are constructed using orthogonal Jacobi polynomials, and the eigenvalue problem is solved via the Rayleigh–Ritz method. Notably, the methodology allows accurate and efficient prediction of axisymmetric (breathing) modes in thick, non-uniform cylindrical shells – a capability rarely addressed in existing literature, despite its importance in Noise, Vibration, and Harshness (NVH) analysis. Validation against Finite Element Analysis (FEA) and Experimental Modal Analysis (EMA) on both generic cylindrical shells and a real Permanent Magnet Synchronous Machine (PMSM) stator shows excellent agreement, with natural frequency deviations typically below 5% and highly consistent mode shapes. The framework also achieves over 95% reduction in computational time compared to FEA, establishing it as a highly adaptable and practical tool for vibration analysis in electric motor design and NVH engineering applications.

## Introduction

In the ever-evolving landscape of Electric Vehicles (EVs), engineers are continuously facing the challenge of optimising and enhancing vehicle performance, reliability, energy efficiency, and passenger comfort. Within the electric (e-) powertrain, a key aspect that can greatly influence the above challenges is Noise, Vibration and Harshness (NVH) [1]. In the absence of the internal combustion engine masking noise, mechanical noises from rolling element bearings and the transmission, as well as aerodynamic noise have become more prominent. However, the most severe contributor of noise in e-powertrains arises from the e-motor, manifesting in the form of a high pitch tonal noise, referred to as whistling [2]. This distinctive phenomenon is a result of the temporal and spatial variation of the electromagnetic stresses generated during the operation of the e-motor. These stresses possess distinct frequency and spatial harmonic characteristics depending on the machine’s topology [3] and are induced at the interface between the airgap and the ferromagnetic material of the stator, causing it to vibrate and radiate airborne noise [4].

Consequently, a thorough understanding of the stator's vibrational behaviour is imperative for accurately predicting and mitigating electromagnetic vibration and noise. In the realm of identifying the modal characteristics and dynamic response of radial flux stators, literature has delineated two primary approaches. The majority of NVH studies employ numerical Finite Element Analysis (FEA) techniques, valued for their high fidelity and precision [5–9]. Conversely, analytical methodologies often resort to oversimplified 2-dimensional (2D) structural models to depict the stator, utilizing equivalent geometrical and material properties to expedite computational processes [10–12]. However, both approaches are not without drawbacks—complex numerical models incur high computational costs, and 2D analytical models rely on critical assumptions. Acknowledging these limitations, the present work introduces an innovative dynamic analysis for radial flux e-motor stators based on the three-dimensional (3D) first-order shear deformation theory for circular cylindrical shells.

The eigenproblem of circular cylindrical shells has been investigated in numerous studies in the literature, due to the abundance of cylindrical shells in engineering practises including pipes, carbon nanotubes, etc. [13–20]. Consequently, a multitude of theories and solution approaches has been developed, with their application being contingent upon the dimensions of the shell and the anticipated magnitude of deformations. In the context of the present study, established guidelines from the literature indicate that, based on the geometry of the stator under examination, shear deformation and rotary inertia effects must be included in the formulation of the free and forced vibration analysis. Consequently, reliance on simplifying assumptions and membrane approximations to further simplify the modelling procedure is precluded.

An earlier study by Naghdi et al. [21], investigated the free vibration of thick shells including the shear deformation and inertia terms in the governing equations, however still relied on some of Love’s thin shell simplifications. This work assumed that displacements and strains in the structure were small relatively to the thickness, and that transverse stress was negligible compared to other stresses, thereby allowing to neglect higher-order strain terms and the transverse stress term, leading to the development of the First-order Shear Deformation Shell Theory (FSDST). Subsequent research by Lim and Liew [22] on thick shallow cylindrical panels extended the inclusion of strain and displacement terms up to the third order, showcasing improvements in predicting natural frequencies for shells of increased thickness. This extended theory is referred to as Higher-order Shear Deformation Shell Theory (HSDST).

More recently, such theories have been utilised to explore more complex geometries and conditions, with functionally graded, non-isotropic materials. Noteworthy examples include the work of Wang et al. [23] whereby the FSDST was employed to investigate the free vibration of a spinning functionally graded spherical–cylindrical-conical shell with arbitrary boundary conditions subjected to a thermal environment, and the work of Hao et al. [24] who investigated nonlinear vibrations in porous conical shells using the generalized differential quadrature method. In another study by Sahmani et al. [25], the HSDST was utilized to develop a non-classical shell model containing an additional internal length scale parameter in order to investigate the length-dependent dynamic stability response of cylindrical micro-shells made of functionally graded materials (FGM).

While these studies include shear deformations and rotary inertia effects, they have not considered the (1 + *z*/*R*) term in the stress resultant equations, which is necessary for the stresses to be integrated on a trapezoidal-like cross-section of a shell element over its thickness, *z* (*R* is the cylinder radius) [26]. This omission, which was observed by both Bert [27] and Chang [28], can lead to numerous errors in the derived equations of thick shells. Studies have demonstrated that the inclusion of this term in FSDST yields more accurate results compared to when using HSDST without accounting for that term [29, 30]. The trapezoidal term was also integrated in the works of Qatu [31] and Thang et al.[32], where the FSDST was used for the free vibration analysis of laminated composite deep thick shells and of bi-directional functionally graded cylindrical shells with varying thicknesses, respectively. Khalili et al. [33] incorporated the trapezoidal term in the HSDST for the free vibration analysis of homogeneous isotropic circular cylindrical shells, while also considering typically neglected effects of transverse shear stresses. Due to the complexity of the mathematical expressions derived through these studies, exact solutions could not be obtained directly, hence approximate solution techniques like the Rayleigh–Ritz and Galerkin methods were implemented to obtain solutions for the eigenvalue problem.

Studies have applied the shear deformation theories for the eigenproblem analysis of stator assemblies, rather than 2D ring approximations, due to the method’s ability to also compute axial and torsional vibrations. In earlier approaches [34, 35], the natural frequencies of laminated stators were predicted based on the 3D theory of elasticity, however with important simplifications and limitations in the boundary conditions. More recently, McCloskey et al. [36] considered the equivalent orthotropic characteristics of the stator assembly in a single-shell approximation. Andreou et al. [37] presented a single-shell approximation method incorporating continuous functions for the characterisation of mechanical property and thickness variations, however the complexity of the functions compromised the computational efficiency of the method. Xing et al. [38], utilised a similar approach with equivalent orthotropic properties for the stator assembly and extended it to predict the steady-state vibration response under electromagnetic excitation. Nonetheless, the combination of all components into one shell with equivalent properties is a significant approximation. Additionally, these studies were limited by boundary conditions. In [39], the stator, including stator core, windings, and casing, were represented as ribbed double cylindrical shells, and the influence of the end covers was introduced by artificial springs. Their results showed a higher prediction accuracy for natural frequencies; however, the authors utilised thin shell approximations which may be unsuitable for such stator assemblies given the dimensions of the system, hence limiting their applicability. Zhao et al. [40] attempted to represent the stator teeth as longitudinal ribs, with the stator modelled as a thick cylindrical shell, again with homogeneous and isotropic material properties. While their approach yielded promising results, it did not account for axisymmetric vibration modes and included limited experimental and numerical validation.

An extension of these methodologies aiming to improve the efficiency and simplify the application of complex boundary conditions and varying material properties involves dividing the shell into a number of segments of potentially distinct material properties and thickness and coupling them using combinations of translational and rotational virtual springs. Such approaches have been readily implemented in the literature, to examine the free and forced vibration behaviours of cylindrical shells, as well as plates, doubly curved shells, and combinations of several different structures. Examples include the work of Zhang et al. [41], who utilised the domain decomposition method to investigate the vibration behaviour of stepped cylindrical shells. Wei et al. [42] employed the same formulation to analyse the generation mechanism of mode localizations in cylindrical shells when connection bolts in the boundaries of the shell loosened. Similarly, Qu et al. [43] introduced a modified variational principle together with a weighted least squares residual approach to enforce the continuity and boundary conditions in circular cylindrical shells. In other studies, Qin et al. [44] and Li et al. [45], proposed unified Jacobi-Ritz formulations for the analysis of different kinds of stepped coupled doubly curved shell structures, whereas Lu et al. [46] applied the method to conical shells, illustrating the versatility of using Jacobi polynomials for the displacement functions of the shells. Nevertheless, although the method offers significant advantages over the single-shell FSDST or HSDST, no studies have implemented it in the analysis of stator assemblies.

Recently, a study by De Barros et al. [47], proposed a semi-analytical method based on shell-element discretisation for fast prediction of stator eigenfrequencies and vibrations in large electrical machines. Their approach demonstrated good agreement with FEA and experimental results, achieving significant computational savings compared to conventional FE models. However, the applicability of their approach was illustrated through a single case study involving one generator model, analysed with and without the stator frame under identical boundary conditions. Additionally, their method relied on a discretised shell mesh with simplified geometrical features, while also neglecting the influence of windings.

In view of the aforementioned findings, this paper presents a novel and comprehensive analytical methodology for the modal analysis of e-motor stator assemblies—a problem that, to the best of the authors' knowledge, has not yet been addressed in such generality and depth in the existing literature. Specifically, this is the first method capable of capturing the effects of housing/cooling jacket, stator teeth, and windings under arbitrary boundary conditions, while also efficiently and accurately predicting both conventional and axisymmetric (breathing) modes. The proposed framework employs the FSDST, with key enhancements including shear deformation effects, rotary inertia, and the inclusion of the trapezoidal stress distribution term, all of which are critical for the accurate representation of thick-walled structures such as stators with high thickness-to-radius ratios. Additionally, the devised method incorporates both continuous and piecewise variations in material properties and thickness through property distribution functions and axial segmentation, for realistic modelling of industrial stator assemblies. The displacement fields of the shell are formulated using well-established orthogonal Jacobi Polynomial functions, and the solution to the eigenvalue problem is obtained by calculating the Lagrangian energy of the system and using the Rayleigh–Ritz approach. The methodology is first validated against FEA models of three thick cylindrical shells with arbitrary material and geometric variations and then applied to a real-world Permanent Magnet Synchronous Machine (PMSM) stator assembly, with excellent agreement achieved against both FE simulations and EMA results. This work thus introduces an accurate, generalisable, and computationally efficient analytical tool for the dynamic characterization of complex e-motor stator assemblies, including breathing modes and represents a significant innovation in the structural analysis and design of modern electric machines.

## Theoretical formulation

### Description of the mechanical model

**Fig. 1:**
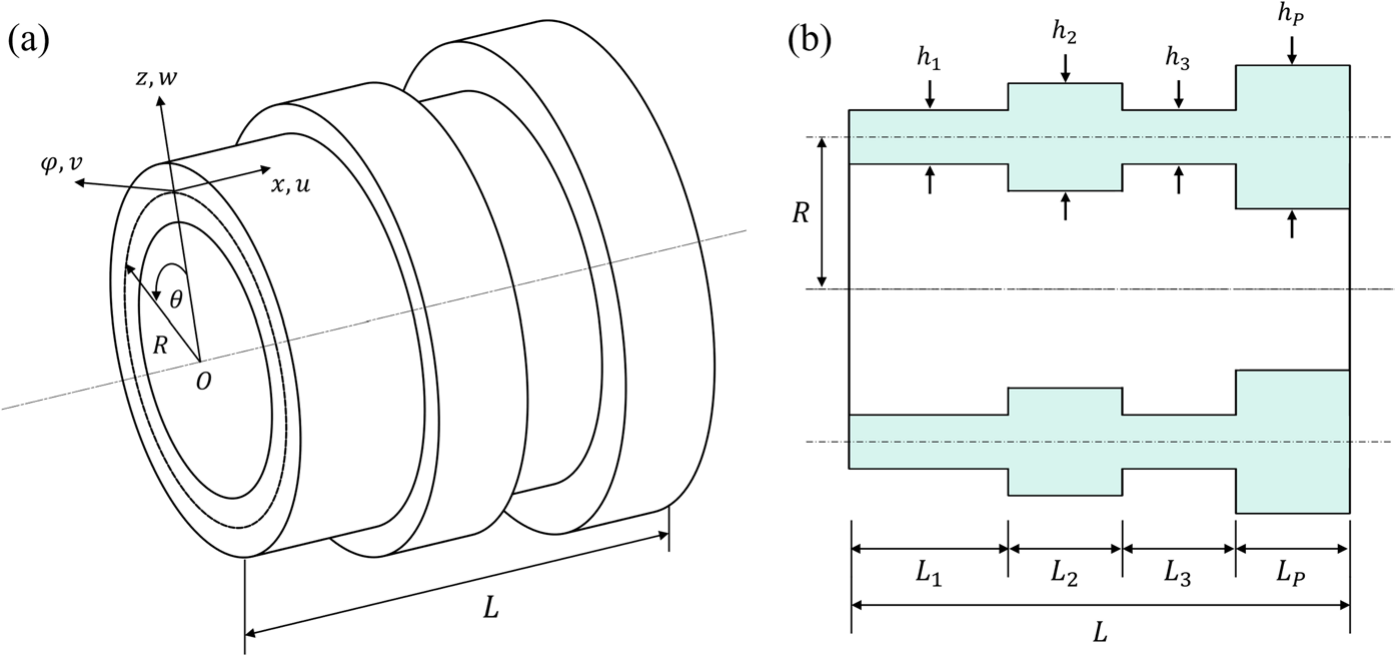
Schematic of a typical thick cylindrical shell of non-uniform thickness: (a) 3D geometry and coordinate system, (b) cross-sectional view of cylindrical shell with $P$ number of segments of length ${\mathrm{L}}_{\mathrm{p}}$ and thickness ${\mathrm{h}}_{\mathrm{p}}$

The mechanical model of the segmented thick circular cylindrical shell is shown in Fig. 1a. The cylindrical coordinate system is placed at the middle surface of the shell, which is at a distance $R$, representing the mean radius of the structure from the origin, $O$. The middle surface displacement components in the axial $x$, tangential $\varphi$, and normal $z$ directions are denoted by $u$, $v$ and $w$, respectively. The total length of the shell is $L$, and it can be segmented according to any number of shell segments $P$, each of length ${L}_{P}$ and thickness ${h}_{P}$, as illustrated in \* MERGEFORMAT Fig. 1b. To utilise the methodology for the modal analysis of such systems, the following assumptions were made [40]:

i. Normal stresses are small relatively to the stresses in the tangential and axial directions, hence shear displacements in the normal direction may be neglected.
ii. Due to the high stiffness of the structure, the displacements are small relatively to its thickness, hence higher order terms in the displacement functions may be neglected, and the use of FSDST is applicable.
iii. Due to the interference fit between the stator and the housing/cooling jacket, there is no relative motion between the two structures at the boundary between them.
iv. The consideration of teeth and winding effects in the analysis is achieved via additional mass on the stator yoke. This assumption has been widely employed in the literature [10], and in the present study is supported by the close agreement between both analytical and numerical predictions against the experimental data. While studies have shown that tooth eigenmodes can influence the vibration behaviour of some electrical machines [48], these are often reported at higher frequencies where increased damping may reduce their contribution to radiated noise. The behaviour of windings has also been shown to depend on manufacturing and impregnation type [49]; however, for the machine studied here their influence on the natural frequencies was assumed negligible.

### Material properties

**Fig. 2:**
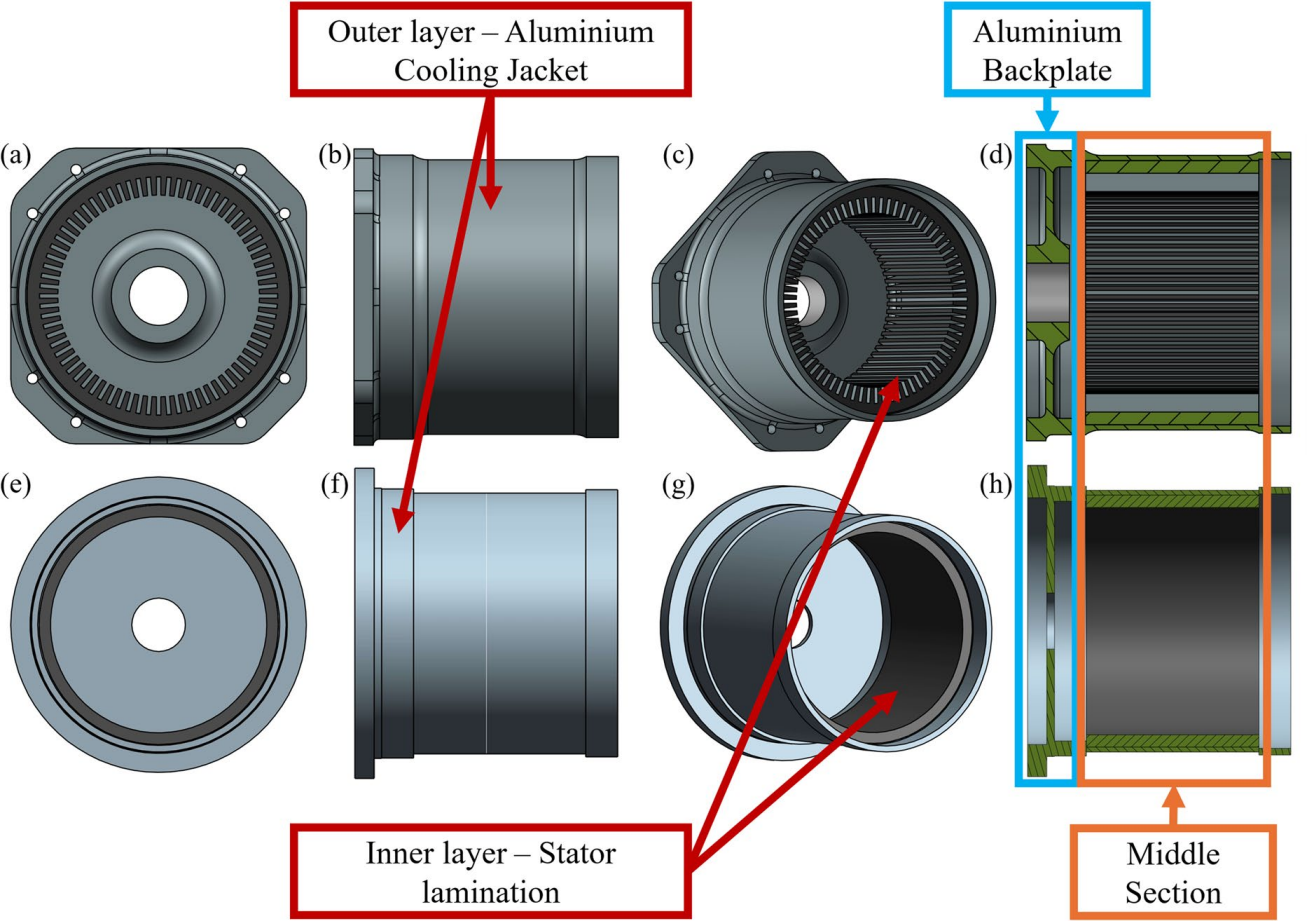
3D geometry of the stator assembly from an e-powertrain, (**a**) rear, (**b**) side, (**c**) trimetric and (**d**) sectional view and equivalent 3D cylindrical shell, (**e**) rear, (**f**) side, (**g**) trimetric and (**h**) sectional view

In this paper, the stator assembly – including the stator core, windings and jacket enclosure – is considered as a single cylindrical shell with mechanical properties that vary along its thickness and non-uniform thickness along its length. The assembly is initially divided into $P$ segments along the axial direction similar to the example in \* MERGEFORMAT Fig. 1, with each segment assigned a mean radius and thickness based on the real geometry, in order to maintain the mass and inertia of the segment. The aluminium cooling jacket backplate used to attach the assembly to the powertrain housing (see Fig. 2), is modelled with isotropic and homogeneous properties. Regarding the middle section, continuous expressions are required to describe the change in mechanical properties between the inner orthotropic layer representing the laminated stator core and the outer isotropic layer representing the aluminium cooling jacket. For the inner layer, equivalent material parameters are determined, using the parameter calculation method of composite materials. The equivalent elastic moduli of the laminated inner layer can be computed for the axial direction following the Reuss series model and the Voigt parallel model for the normal and tangential directions [2, 50, 51] as shown in Eqs. (1)–(2). These models assume uniform stress and strain conditions, and do not explicitly account for interlaminar contact conditions or clamping pressure, which have been shown to affect the equivalent axial properties of laminated stacks [52]. In the present study, their use was deemed sufficient to characterise the orthotropic behaviour of the stator core, but the resulting properties should be interpreted as approximations whose accuracy depends on the lamination assembly conditions.

1 $${{E}_{\mathrm{inner}}}_{x}={\left(\frac{{V}_{s}}{{E}_{s}}+\frac{{V}_{l}}{{E}_{l}}\right)}^{-1}$$

2 $${{E}_{\mathrm{inner}}}_{z}={{E}_{\mathrm{inner}}}_{\varphi }={V}_{s}{E}_{s}+{V}_{l}{E}_{l}$$

where ${E}_{s}$ and ${E}_{l}$ are respectively the elastic moduli of the silicon steel and the lamination material while ${V}_{s}$ and ${V}_{l}$ are their respective volume fractions. Here, ${V}_{s}$ is equivalent to the stator lamination factor taken to be $0.96$ [12], hence ${V}_{l}=1- {V}_{s}=0.04$. All the materials are assumed to have the same Poisson’s ratio, $\mu$. Shear moduli, $G$ of the transversely isotropic inner layer representing the stator core have been determined using Eqs. (3) and (4) [39, 53] and the equivalent density, $\rho$ has been calculated through Eq. (5), by modifying the volume mixture rule [54] to include the masses of the teeth and the windings, ${m}_{w}$. The parameters used for the calculation of the equivalent material properties are provided in Table 1.

**Table 1:** Nominal material parameters used for calculating the stator’s orthotropic properties

Parameter		Value
Silicon steel elastic modulus	${E}_{s}$	$206\text{ GPa}$
Lamination elastic modulus	${E}_{l}$	$3\text{ GPa}$
Poisson’s ratio	$\mu$	$0.3$
Silicon steel density	${\rho }_{s}$	$7600\text{ kg}/{\mathrm{m}}^{3}$
Lamination density	${\rho }_{l}$	$1300\text{ kg}/{\mathrm{m}}^{3}$
Teeth volume	${V}_{\mathrm{teeth}}$	$5.79\times {10}^{-4} {\mathrm{m}}^{3}$
Winding mass	${m}_{w}$	$3.63\text{ kg}$

3 $${G}_{\mathrm{inne}{r}_{z\varphi }}=\frac{{{E}_{\mathrm{inner}}}_{z}}{2\left(1+\mu \right)}$$

4 $${{G}_{\mathrm{inner}}}_{x\varphi }= {{G}_{\mathrm{inner}}}_{xz}=1/\left(\left(\frac{{V}_{s}}{{G}_{s}}\right)+\left(\frac{{V}_{l}}{{G}_{l}}\right)\right)$$

5 $${\rho }_{\mathrm{equivalent}}=\frac{{V}_{s}{\rho }_{s}+ {V}_{l}{\rho }_{l}+\left(\frac{{V}_{\mathrm{teeth}}\times \left({V}_{s}{\rho }_{s}+ {V}_{l}{\rho }_{l}\right)}{\left({V}_{s}+ {V}_{l}\right)}\right)+{m}_{w}}{{V}_{\mathrm{inner}}}$$

Having calculated the above parameters, a set of continuous expressions has been devised to describe the variation of the continuous equivalent elastic modulus, shear modulus and density within the cylindrical shell, in the form of:

6 $${E}_{x}\left(z\right)=\left[\left({E}_{{\mathrm{outer}}_{x}}-{E}_{{\mathrm{inner}}_{x}}\right)\times \mathcal{H}\left(\frac{z}{{h}_{p}}\right)\right]+ {E}_{{\mathrm{inner}}_{x}}$$

7 $${E}_{z}\left(z\right)={E}_{\varphi }\left(z\right)=\left[\left({E}_{{\mathrm{outer}}_{z}}-{E}_{{\mathrm{inner}}_{z}}\right)\times \mathcal{H}\left(\frac{z}{{h}_{p}}\right)\right]+ {E}_{{\mathrm{inner}}_{z}}$$

8 $${G}_{z\varphi }\left(z\right)=\left[\left({G}_{{\mathrm{outer}}_{z\varphi }}-{G}_{{\mathrm{inner}}_{z\varphi }}\right)\times \mathcal{H}\left(\frac{z}{{h}_{p}}\right)\right]+ {G}_{{\mathrm{inner}}_{z\varphi }}$$

9 $${G}_{xz}\left(z\right)={G}_{x\varphi }\left(z\right)=\left[\left({G}_{{\mathrm{outer}}_{xz}}-{G}_{{\mathrm{inner}}_{xz}}\right)\times \mathcal{H}\left(\frac{z}{{h}_{p}}\right)\right]+ {G}_{{\mathrm{inner}}_{xz}}$$

10 $$\rho \left(z\right)=\left[\left({\rho }_{\mathrm{outer}}-{\rho }_{\mathrm{inner}}\right)\times \mathcal{H}\left(\frac{z}{{h}_{p}}\right)\right]+ {\rho }_{\mathrm{inner}}$$

Here $\mathcal{H}\left(z\right)$ represents the Heaviside Theta function, which was selected for this method due to its ability to introduce sharp changes in the material properties and accurately represent the transition from one layer to the other.

### Energy functional for thick circular cylindrical shells

Assuming that the shell’s displacements are small relative to its thickness and that normal shear stresses and deformations are negligible, the FSDST may be applied on modelling the structure [31]. The displacements at any location and time $t$, can be expressed as:

11 $${u}^{p}\left(x,\varphi ,z,t\right)= {u}_{0}^{p}\left(x,\varphi ,t\right)+z{\beta }_{x}^{p}(x,\varphi ,t)$$

12 $${v}^{p}\left(x,\varphi ,z,t\right)= {v}_{0}^{p}\left(x,\varphi ,t\right)+z{\beta }_{\varphi }^{p}(x,\varphi ,t)$$

13 $${w}^{p}\left(x,\varphi ,z,t\right)= {w}_{0}^{p}\left(x,\varphi ,t\right)$$

where ${u}_{0}^{p}$, ${v}_{0}^{p}$ and ${w}_{0}^{p}$ are the mid-surface displacements of the $p$-th segment in the axial, circumferential and normal directions, and ${\beta }_{x}^{p}$ and ${\beta }_{\varphi }^{p}$ are the mid-surface rotations in the axial and circumferential directions, respectively. According to the linear theory of elasticity, the strain components at any point on the $p$-th segment may be expressed as [29]:

14 $${\varepsilon }_{x}^{p}={\varepsilon }_{x}^{0,p}+z{\kappa }_{x}^{p}$$

15 $${\varepsilon }_{\varphi }^{p}=\left({\varepsilon }_{\varphi }^{0,p}+z{\kappa }_{\varphi }^{p}\right)\left(\frac{1}{1+z/R}\right)$$

16 $${\varepsilon }_{x\varphi }^{p}={\varepsilon }_{x\varphi }^{0,p}+z{\kappa }_{x\varphi }^{p}$$

17 $${\varepsilon }_{\varphi x}^{p}=\left({\varepsilon }_{\varphi x}^{0,p}+z{\kappa }_{\varphi x}^{p}\right)\left(\frac{1}{1+z/R}\right)$$

18 $${\gamma }_{x\varphi }^{p}= {\varepsilon }_{x\varphi }^{p} + {\varepsilon }_{\varphi x}^{p}$$

19 $${\gamma }_{xz}^{p}= {\gamma }_{xz}^{0, p}$$

20 $${\gamma }_{\varphi z}^{p}=\left({\gamma }_{\varphi z}^{0, p}\right)\left(\frac{1}{1+z/R}\right)$$

where ${\varepsilon }_{x}^{0,p}$, ${\varepsilon }_{\varphi }^{0,p}$, ${\varepsilon }_{x\varphi }^{0,p}$, ${\varepsilon }_{\varphi x}^{0,p}$, ${\gamma }_{xz}^{0, p}$ and ${\gamma }_{\varphi z}^{0, p}$ are the mid-surface strains and ${\kappa }_{x}^{p}$, ${\kappa }_{\varphi }^{p}$, ${\kappa }_{x\varphi }^{p}$ and ${\kappa }_{\varphi x}^{p}$ are the mid-surface curvatures of the $p$-th segment. These are defined by:

21 $${\varepsilon }_{x}^{0,p}=\frac{\partial {u}_{0}^{p}}{\partial x}$$

22 $${\varepsilon }_{\varphi }^{0,p}=\frac{1}{R}\left(\frac{\partial {v}_{0}^{p}}{\partial \varphi }+{w}_{0}^{p}\right)$$

23 $${\varepsilon }_{x\varphi }^{0,p}= \frac{\partial {v}_{0}^{p}}{\partial x}$$

24 $${\varepsilon }_{\varphi x}^{0,p}=\frac{1}{R}\frac{\partial {u}_{0}^{p}}{\partial \varphi }$$

25 $${\gamma }_{xz}^{0, p}= \frac{\partial {w}_{0}^{p}}{\partial x}+{\beta }_{x}^{p}$$

26 $${\gamma }_{\varphi z}^{0, p}=\frac{1}{R}\frac{\partial {w}_{0}^{p}}{\partial \varphi }-\frac{{v}_{0}^{p}}{R}+{\beta }_{\varphi }$$

27 $${\kappa }_{x}^{p}= \frac{\partial {\beta }_{x}}{\partial x}$$

28 $${\kappa }_{\varphi }^{p}=\frac{1}{R}\frac{\partial {\beta }_{\varphi }}{\partial \varphi }$$

29 $${\kappa }_{x\varphi }^{p}= \frac{\partial {\beta }_{\varphi }}{\partial x}$$

30 $${\kappa }_{\varphi x}^{p}=\frac{1}{R}\frac{\partial {\beta }_{x}}{\partial \varphi }$$

According to the generalized Hooke’s law, stress–strain relationships for orthotropic bodies are derived by [55]:

31 $$\left[\begin{array}{c}{\sigma }_{xx}^{p}\\ {\sigma }_{\varphi \varphi }^{p}\\ {\tau }_{x\varphi }^{p}\\ {\tau }_{xz}^{p}\\ {\tau }_{\varphi z}^{p}\end{array}\right]= \left[\begin{array}{ccccc}{Q}_{11}^{p}(z)& {Q}_{12}^{p}(z)& 0& 0& 0\\ {Q}_{21}^{p}(z)& {Q}_{22}^{p}(z)& 0& 0& 0\\ 0& 0& {Q}_{33}^{p}(z)& 0& 0\\ 0& 0& 0& {Q}_{44}^{p}(z)& 0\\ 0& 0& 0& 0& {Q}_{55}^{p}(z)\end{array}\right].\left[\begin{array}{c}{\varepsilon }_{x}^{p}\\ {\varepsilon }_{\varphi }^{p}\\ {\gamma }_{x\varphi }^{p}\\ {\gamma }_{xz}^{p}\\ {\gamma }_{\varphi z}^{p}\end{array}\right]$$

where $\sigma$ denotes normal stress and $\tau$ denotes shear stress. ${Q}_{ij}^{p}$ are the elastic stiffness coefficients of the material ($i,j=1, 2, 3, 4, 5$) and can be evaluated separately for each shell segment by [55]:

32 $${Q}_{11}^{p}\left(z\right)=\frac{{E}_{x}^{p}\left(z\right)}{1-{\mu }^{2}} , {Q}_{12}^{p}\left(z\right)=\frac{\mu {E}_{\varphi }^{p}\left(z\right)}{1-{\mu }^{2}} , {Q}_{21}^{p}\left(z\right)= \frac{\mu {E}_{x}^{p}\left(z\right)}{1-{\mu }^{2}} , {Q}_{22}^{p}\left(z\right)= \frac{{E}_{\varphi }^{p}\left(z\right)}{1-{\mu }^{2}}$$

33 $${Q}_{33}^{p}\left(z\right)={G}_{z\varphi }^{p}\left(z\right) , {Q}_{44}^{p}\left(z\right)={G}_{x\varphi }^{p}\left(z\right), {Q}_{55}^{p}\left(z\right)={G}_{xz}^{p}\left(z\right)$$

The expression for the strain energy of each shell component, ${U}^{p}$ may then be written as [33]:

34 $${U}^{p}=\frac{R}{2}{\int }_{0}^{{L}_{p}}{\int }_{-{h}_{p}/2}^{{h}_{p}/2}{\int }_{0}^{2\pi }\left({\sigma }_{xx}^{p}{\varepsilon }_{x}^{p}+ {\sigma }_{\varphi \varphi }^{p}{\varepsilon }_{\varphi }^{p}+ {\tau }_{x\varphi }^{p}{\gamma }_{x\varphi }^{p}+ {\tau }_{xz}^{p}{\gamma }_{xz}^{p}+ {\tau }_{\varphi z}^{p}{\gamma }_{\varphi z}^{p}\right)\left(1+\frac{z}{R}\right)d\varphi dzdx$$

and the kinetic energy of each shell segment, ${T}^{p}$ is defined as:

35 $${T}^{p}=\frac{R}{2}{\int }_{0}^{{L}_{p}}{\int }_{-{h}_{p}/2}^{{h}_{p}/2}{\int }_{0}^{2\pi }{\rho }_{p}\left({\dot{u}}_{p}^{2}+ {\dot{v}}_{p}^{2}+ {\dot{w}}_{p}^{2}\right)\left(1+\frac{z}{R}\right)d\varphi dzdx$$

where ${\rho }_{p}$ is the density of the $p$-th shell segment and is a function of $z$, and the superimposed dots denote the time derivative of the shell segment displacements ${u}_{p}$, ${v}_{p}$ and ${w}_{p}$. Finally, the virtual work done by external loads acting on each shell segment can be defined by:

36 $${W}^{p}=\frac{R}{2}{\int }_{0}^{{L}_{p}}{\int }_{-{h}_{p}/2}^{{h}_{p}/2}{\int }_{0}^{2\pi }({f}_{u}^{p}{u}_{0}^{p}+ {f}_{v}^{p}{v}_{0}^{p}+ {f}_{w}^{p}{w}_{0}^{p}+ {f}_{{\beta }_{x}}^{p}{\beta }_{x}^{p}+ {f}_{{\beta }_{\varphi }}^{p}{\beta }_{\varphi }^{p})\left(1+\frac{z}{R}\right)d\varphi dzdx$$

where ${f}_{j}^{p}$ ($j=u, v, w, {\beta }_{x}, {\beta }_{\varphi }$) represent the external loads acting on the $p$-th shell segment in the corresponding direction. Integrals (34)—(36) were evaluated symbolically using Wolfram Mathematica. The closed-form expressions are not reproduced here due to their length and case-specific dependence on the Heaviside theta functions used for property definitions. However, all integrals are solved analytically before substitution of numerical parameters.

#### Boundary and continuity constraints

**Fig. 3:**
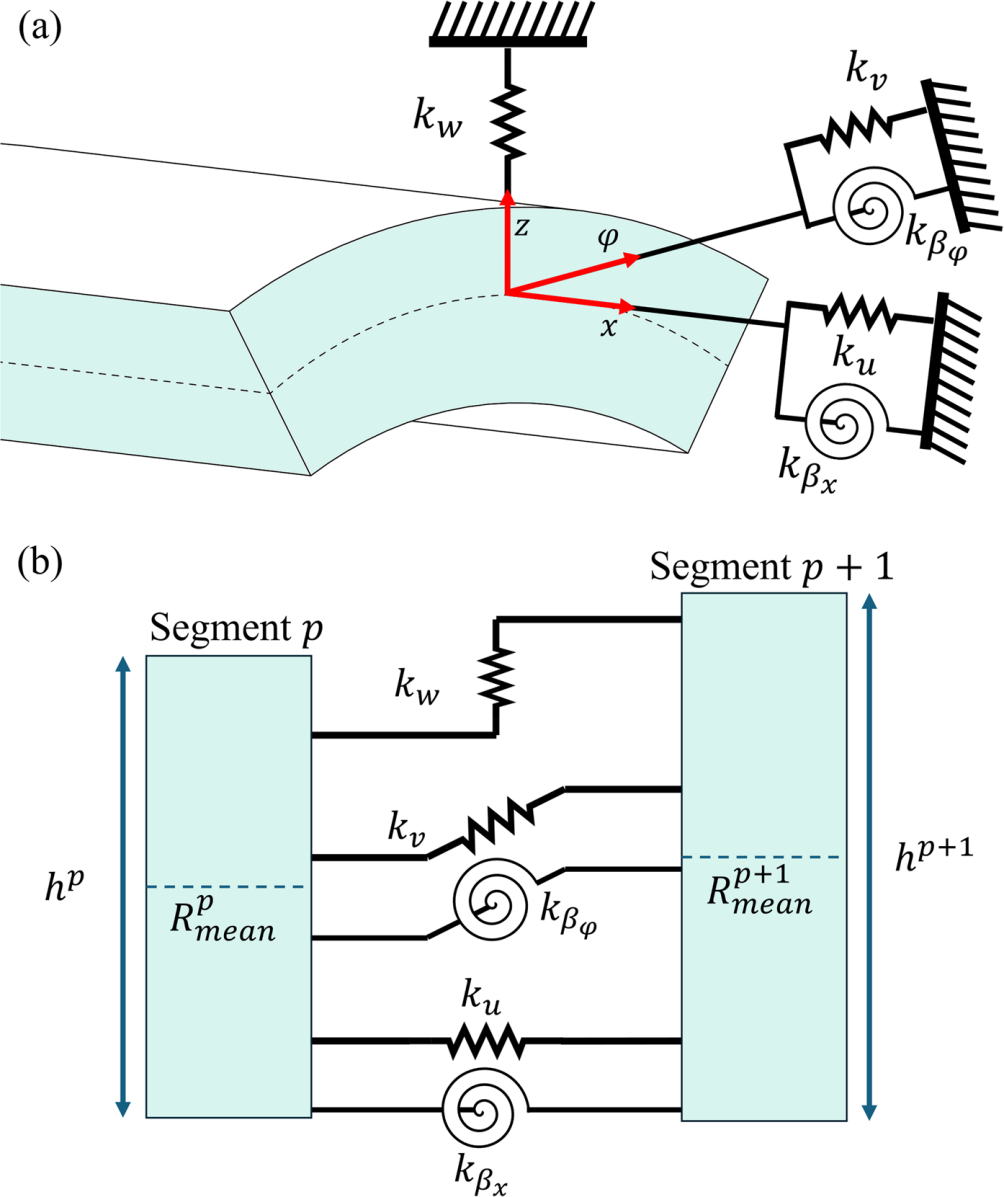
**a** Schematic of artificial spring arrangement used to implement different boundary conditions at the edge of the cylinder and **b**, schematic of artificial spring arrangement implemented between adjacent shell segments $p$ and $p+1$ to impose continuity

In this study, the penalty method [56] was utilised for the application of appropriate constraints at the ends of each shell segment, such that correct boundary conditions and continuity between the transverse and shear displacements of consecutive segments is ensured. This is illustrated in Fig. 3, with three linear springs and two torsional springs, as a torsional spring for normal mid-surface rotations in the normal direction is redundant based on assumption (i) in Sect. 2.1. Additionally, by varying the stiffness of the springs used at either end of the cylindrical shell, a variety of boundary conditions may be easily introduced. The energy stored in the boundary springs, ${U}_{b}$ is defined as:

37 $${U}_{b}={U}_{b}^{0}+{U}_{b}^{L}$$

where:

38 $${U}_{b}^{0}=\frac{R}{2}{\int }_{-\frac{{h}_{1}}{2}}^{\frac{{h}_{1}}{2}}{{\int }_{0}^{2\pi }\left\{{k}_{u}^{0}{\left({u}_{0}^{1}\right)}^{2}+ {k}_{v}^{0}{\left({v}_{0}^{1}\right)}^{2}+ {k}_{w}^{0}{\left({w}_{0}^{1}\right)}^{2}+ {k}_{{\beta }_{x}}^{0}{\left({\beta }_{x}^{1}\right)}^{2}+ {k}_{{\beta }_{\varphi }}^{0}{\left({\beta }_{\varphi }^{1}\right)}^{2} \right\}}_{x=0}\left(1+\frac{z}{R}\right)d\varphi dz$$

39 $${U}_{b}^{L}=\frac{R}{2}{\int }_{-\frac{{h}_{P}}{2}}^{\frac{{h}_{P}}{2}}{{\int }_{0}^{2\pi }\left\{{k}_{u}^{L}{\left({u}_{0}^{P}\right)}^{2}+ {k}_{v}^{L}{\left({v}_{0}^{P}\right)}^{2}+ {k}_{w}^{L}{\left({w}_{0}^{P}\right)}^{2}+ {k}_{{\beta }_{x}}^{L}{\left({\beta }_{x}^{P}\right)}^{2}+ {k}_{{\beta }_{\varphi }}^{L}{\left({\beta }_{\varphi }^{P}\right)}^{2} \right\}}_{x=L}\left(1+\frac{z}{R}\right)d\varphi dz$$

Constants ${k}_{j}^{0}$ ($j=u, v, w, {\beta }_{x}, {\beta }_{\varphi }$) and ${k}_{j}^{P}$ represent the spring coefficients at the two sides of the circular cylindrical shell, respectively. Similarly, for adjacent segments, the energy stored in the connective springs is:

40 $${U}_{c}^{p, p+1}=\frac{R}{2}{\int }_{-\frac{{h}_{1}}{2}}^{\frac{{h}_{1}}{2}}{{\int }_{0}^{2\pi }\left\{{k}_{u}{\left({u}_{0}^{p}-{u}_{0}^{p+1}\right)}^{2}+ {k}_{v}{\left({v}_{0}^{p}-{v}_{0}^{p+1}\right)}^{2}+ {k}_{w}{\left({w}_{0}^{p}-{w}_{0}^{p+1}\right)}^{2}+ {k}_{{\beta }_{x}}{\left({\beta }_{x}^{p}-{\beta }_{x}^{p+1}\right)}^{2}+ {k}_{{\beta }_{\varphi }}{\left({\beta }_{\varphi }^{p}-{\beta }_{\varphi }^{p+1}\right)}^{2}+ \right\}}_{x={L}_{p}}\left(1+\frac{z}{R}\right)d\varphi dz$$

### Displacement functions for thick cylindrical shell

The selection of admissible functions to describe the oscillations of the cylinder surface is a key factor in ensuring the convergence and accuracy of the response. Several studies [43–45, 57] have illustrated how the use of Jacobi orthogonal polynomials is highly suitable for this purpose, hence the displacement components of each shell segment are defined in this study as:

41 $${u}_{0}^{p}\left({\overline{x} }^{p},\varphi ,t\right)= \sum_{m=0}^{M}\sum_{n=1}^{N}{U}_{mn}^{p}\left(t\right) {P}_{m}^{\left(\alpha ,\beta \right)}({\overline{x} }^{p})\mathrm{cos}(n\varphi -\theta )$$

42 $${v}_{0}^{p}\left({\overline{x} }^{p},\varphi ,t\right)= \sum_{m=0}^{M}\sum_{n=1}^{N}{V}_{mn}^{p}\left(t\right) {P}_{m}^{\left(\alpha ,\beta \right)}({\overline{x} }^{p})\mathrm{sin}(n\varphi -\theta )$$

43 $${w}_{0}^{p}\left({\overline{x} }^{p},\varphi ,t\right)= \sum_{m=0}^{M}\sum_{n=1}^{N}{W}_{mn}^{p}\left(t\right) {P}_{m}^{\left(\alpha ,\beta \right)}({\overline{x} }^{p})\mathrm{cos}(n\varphi -\theta )$$

44 $${\beta }_{x}^{p}\left({\overline{x} }^{p},\varphi ,t\right)= \sum_{m=0}^{M}\sum_{n=1}^{N}{{\rm B}_{x}}_{mn}^{p}\left(t\right) {P}_{m}^{\left(\alpha ,\beta \right)}({\overline{x} }^{p})\mathrm{cos}(n\varphi -\theta )$$

45 $${\beta }_{\varphi }^{p}\left({\overline{x} }^{p},\varphi ,t\right)= \sum_{m=0}^{M}\sum_{n=1}^{N}{{\rm B}_{\varphi }}_{mn}^{p}\left(t\right) {P}_{m}^{\left(\alpha ,\beta \right)}({\overline{x} }^{p})\mathrm{sin}(n\varphi -\theta )$$

In Eqs. (41)–(45), ${P}_{m}^{\left(\alpha ,\beta \right)}({\overline{x} }^{p})$ are the ${m}^{th}$ order Jacobi polynomial functions describing the displacement in the axial direction and are defined in Eq. (46) [58]. Coefficients $m$ and $n$ correspond to the axial and circumferential wave number with the highest degree of $M$ and $N$, respectively, while ${U}_{mn}^{p}\left(t\right)$, ${V}_{mn}^{p}\left(t\right)$, ${W}_{mn}^{p}\left(t\right)$, ${{B}_{x}}_{mn}^{p}\left(t\right)$ and ${{B}_{\varphi }}_{mn}^{p}\left(t\right)$ are the generalized coordinate variables for the $p$-th shell segment, corresponding to the cylindrical mode $(m,n)$. Coefficient $\theta$ corresponds to the vibration mode phase angle, which for free, undamped oscillations of axisymmetric structures has the value of $0$ and $\uppi /(2n)$ for orthogonal vibration modes [59]

46 $${P}_{m}^{\left(\alpha ,\beta \right)}\left({\overline{x} }^{p}\right)=\left\{\begin{array}{c}1 , m=0\\ \frac{\alpha +\beta +2}{2}{\overline{x} }^{p} -\frac{\alpha -\beta }{2} , m=1\\ \frac{\left(\alpha +\beta +2m-1\right)\left\{{\alpha }^{2}-{\beta }^{2}+{\overline{x} }^{p}\left(\alpha +\beta +2m\right)\left(\alpha +\beta +2m-2\right)\right\}}{2m\left(\alpha +\beta +m\right)\left(a+\beta +2m-2\right)}{P}_{m-1}^{\left(\alpha ,\beta \right)}\left({\overline{x} }^{p}\right) -\frac{\left(\alpha +m-1\right)\left(\beta +m-1\right)\left(\alpha +\beta +2m\right)}{m\left(\alpha +\beta +m\right)(a+b+2\mu -2)}{P}_{m-2}^{\left(\alpha ,\beta \right)}\left({\overline{x} }^{p}\right), m\ge 2\end{array}\right.$$

where $\alpha , \beta>-1$. By setting $\alpha =\beta =-1/2$, the Chebyshev polynomials of the first kind are obtained; $\alpha = \beta =1/2$ leads to the Chebyshev polynomials of the second kind and by setting $\alpha =\beta =0$, one obtains the Legendre polynomials. However, the effect of changing these coefficients is trivial as shown by [45], as long as $\alpha =\beta$. It must be noted that the above polynomial functions are all orthogonal and are defined in the range of $\overline{x }\in [-\mathrm{1,1}]$. Hence, to allow for their use in this approach, a linear coordinate transformation is introduced from the interval $x\in [{L}_{p},{L}_{p+1}]$ of the $p$-th shell segment to $\overline{x }\in [-\mathrm{1,1}]$, as shown below:

47 $${\overline{x} }^{p}=\frac{x-[\left({L}_{p+1}+{L}_{p}\right)/2]}{\left({L}_{p+1}-{L}_{p}\right)/2}$$

**Fig. 4:**
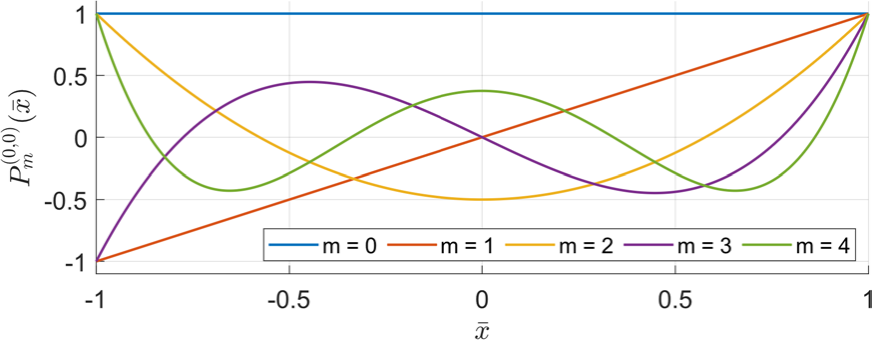
Jacobi polynomial functions ${P}_{m}^{\left(\mathrm{0,0}\right)}(\overline{x })$ of orders $m=0-4$, plotted over the normalized axial coordinate $\overline{x }\in [-\mathrm{1,1}]$

Figure 4 illustrates several of the Jacobi polynomial functions used in Eq. (46) for increasing orders $m$. In the Ritz formulation these polynomials act as admissible functions for the axial coordinate, and appropriate linear combinations of them reconstruct the axial distribution of each cylindrical mode shape. This enables the method to represent both global bending modes (dominated by low-order terms) and localized higher-order axial variations. By substituting Eqs. (46) and (47) into Eqs. (41)–(45), and subsequently into Eqs. (34)–(40), the strain, kinetic, boundary and connective spring energies and the virtual work done by external loads may be evaluated. The total Lagrangian energy function, $\mathcal{L}$ may be thus expressed as:

48 $$\mathcal{L}= \sum_{p=1}^{P}\left({T}^{p}-{U}^{p}+{W}^{p}\right)- \sum_{p=1}^{P-1}{U}_{c}^{p, p+1}-{U}_{b}$$

According to the Rayleigh–Ritz formulation [59], by minimising the Lagrangian energy function with respect to each of the generalised coordinate variables, ${U}_{mn}^{p}$, ${V}_{mn}^{p}$, ${W}_{mn}^{p}$, ${{\rm B}_{x}}_{mn}^{p}$ and ${{\rm B}_{\varphi }}_{mn}^{p}$, the equations of motion for a linear and undamped stepped circular cylindrical shell may be determined as:

49 $${\boldsymbol{M}}\ddot{{\boldsymbol{q}}}+{\boldsymbol{K}}{\boldsymbol{q}}={\boldsymbol{F}}$$

where ${\boldsymbol{q}}$ is the global generalised coordinate vector, ${\boldsymbol{M}}$ and ${\boldsymbol{K}}$ are respectively the disjoint generalised mass and stiffness matrices and ${\boldsymbol{F}}$ is the external excitation vector.

### Eigenproblem analysis

Assuming no external work and that the system undergoes free harmonic motion, i.e. ${\boldsymbol{F}}=0$ and ${\boldsymbol{q}}({\boldsymbol{t}})=\overline{{\boldsymbol{q}}}{e }^{i\omega t}$, Eq. (48) may be written as

50 $$[-{\omega }_{mn}^{2}{\boldsymbol{M}}+{\boldsymbol{K}}]\overline{{\boldsymbol{q}} }=0$$

The solution of the eigenvalue problem produces the natural frequencies, ${\omega }_{nat}$ and corresponding eigenvectors, $\overline{{\boldsymbol{q}} }$. The mode shapes of the system may be reconstructed for each natural frequency, by substituting the respective eigenvector back into Eqs. (41)–(45).

### Axisymmetric vibration modes

In the special case of axisymmetric modes when $n=0$, the circumferential dependence is absent, as all the points in the circumference of the cylinder at any location along its length expand and contract simultaneously at the same rate. Hence, it can be easily identified that Eqs. (41)−(45) become:

51 $${u}_{0}^{p}\left({\overline{x} }^{p},t\right)= \sum_{m=0}^{M}\sum_{n=1}^{N}{U}_{mn}^{p}\left(t\right) {P}_{m}^{\left(\alpha ,\beta \right)}({\overline{x} }^{p})$$

52 $${v}_{0}^{p}\left({\overline{x} }^{p},t\right)= 0$$

53 $${w}_{0}^{p}\left({\overline{x} }^{p},t\right)= \sum_{m=0}^{M}\sum_{n=1}^{N}{W}_{mn}^{p}\left(t\right) {P}_{m}^{\left(\alpha ,\beta \right)}({\overline{x} }^{p})$$

54 $${\beta }_{x}^{p}\left({\overline{x} }^{p},t\right)= \sum_{m=0}^{M}\sum_{n=1}^{N}{{\rm B}_{x}}_{mn}^{p}\left(t\right) {P}_{m}^{\left(\alpha ,\beta \right)}({\overline{x} }^{p})$$

55 $${\beta }_{\varphi }^{p}\left({\overline{x} }^{p},t\right)= 0$$

as also shown in [60], suggesting that the displacement field firstly becomes purely a function of $\overline{x }$, and secondly that tangential transverse and shear displacements become trivial. Utilising these observations, the displacements of any point and at any time $t$, may be expressed as:

56 $${u}^{p}\left({\overline{x} }^{p},z,t\right)= {u}_{0}^{p}\left({\overline{x} }^{p},t\right)+z{\beta }_{x}^{p}({\overline{x} }^{p},t)$$

57 $${w}^{p}\left({\overline{x} }^{p},z,t\right)= {w}_{0}^{p}\left({\overline{x} }^{p},t\right)$$

and the strain components as:

58 $${\varepsilon }_{x}^{p}={\varepsilon }_{x}^{0,p}+z{\kappa }_{x}^{p}$$

59 $${\varepsilon }_{\varphi }^{p}=\left({\varepsilon }_{\varphi }^{0,p}\right)\left(\frac{1}{1+z/R}\right)$$

60 $${\gamma }_{xz}^{p}= {\gamma }_{xz}^{0, p}$$

where the mid-surface strains and curvatures now become:

61 $${\varepsilon }_{x}^{0,p}=\frac{\partial {u}_{0}^{p}}{\partial x}$$

62 $${\varepsilon }_{\varphi }^{0,p}=\frac{1}{R}\left({w}_{0}^{p}\right)$$

63 $${\gamma }_{xz}^{0, p}= \frac{\partial {w}_{0}^{p}}{\partial x}+{\beta }_{x}^{p}$$

64 $${\kappa }_{x}^{p}= \frac{\partial {\beta }_{x}}{\partial x}.$$

Hence, the strain energy of each shell segment may now be calculated by:

65 $${U}^{p}=\frac{R}{2}{\int }_{0}^{{L}_{p}}{\int }_{-{h}_{p}/2}^{{h}_{p}/2}{\int }_{0}^{2\pi }\left({\sigma }_{xx}^{p}{\varepsilon }_{x}^{p}+ {\sigma }_{\varphi \varphi }^{p}{\varepsilon }_{\varphi }^{p}+ {\tau }_{xz}^{p}{\gamma }_{xz}^{p}\right)\left(1+\frac{z}{R}\right)d\varphi dzdx$$

and the kinetic energy as:

66 $${T}^{p}=\frac{R}{2}{\int }_{0}^{{L}_{p}}{\int }_{-{h}_{p}/2}^{{h}_{p}/2}{\int }_{0}^{2\pi }{\rho }_{p}\left({\dot{u}}_{p}^{2}+ {\dot{w}}_{p}^{2}\right)\left(1+\frac{z}{R}\right)d\varphi dzdx$$

Finally, by re-evaluating the energy stored in the artificial boundary and continuity springs through:

67 $${U}_{b}^{0}=\frac{R}{2}{\int }_{-\frac{{h}_{1}}{2}}^{\frac{{h}_{1}}{2}}{{\int }_{0}^{2\pi }\left\{{k}_{u}^{0}{\left({u}_{0}^{1}\right)}^{2}+ {k}_{w}^{0}{\left({w}_{0}^{1}\right)}^{2}+ {k}_{{\beta }_{x}}^{0}{\left({\beta }_{x}^{1}\right)}^{2} \right\}}_{x=0}\left(1+\frac{z}{R}\right)d\varphi dz$$

68 $${U}_{b}^{L}=\frac{R}{2}{\int }_{-\frac{{h}_{P}}{2}}^{\frac{{h}_{P}}{2}}{{\int }_{0}^{2\pi }\left\{{k}_{u}^{L}{\left({u}_{0}^{P}\right)}^{2}+ {k}_{w}^{L}{\left({w}_{0}^{P}\right)}^{2}+ {k}_{{\beta }_{x}}^{L}{\left({\beta }_{x}^{P}\right)}^{2} \right\}}_{x=L}\left(1+\frac{z}{R}\right)d\varphi dz$$

69 $${U}_{c}^{p, p+1}=\frac{R}{2}{\int }_{-\frac{{h}_{1}}{2}}^{\frac{{h}_{1}}{2}}{{\int }_{0}^{2\pi }\left\{{k}_{u}{\left({u}_{0}^{p}-{u}_{0}^{p+1}\right)}^{2}+ {k}_{w}{\left({w}_{0}^{p}-{w}_{0}^{p+1}\right)}^{2}+ {k}_{{\beta }_{x}}{\left({\beta }_{x}^{p}-{\beta }_{x}^{p+1}\right)}^{2} \right\}}_{x={L}_{p}}\left(1+\frac{z}{R}\right)d\varphi dz$$

The Rayleigh–Ritz method, as previously outlined, can be employed to solve Eq. (51) for the axisymmetric natural frequencies and mode shapes of the thick circular cylindrical shell under any boundary conditions. Unlike [60], the method presented in this study enables a significantly more direct and computationally efficient identification of axisymmetric vibration modes, particularly in systems with complex material property distributions and boundary conditions, as it eliminates the need for domain discretization or meshing, unlike [47]. As a result, the proposed approach provides a faster, more insightful, and highly practical tool for the modal analysis of complex cylindrical structures.

## Results and discussion

**Fig. 5:**
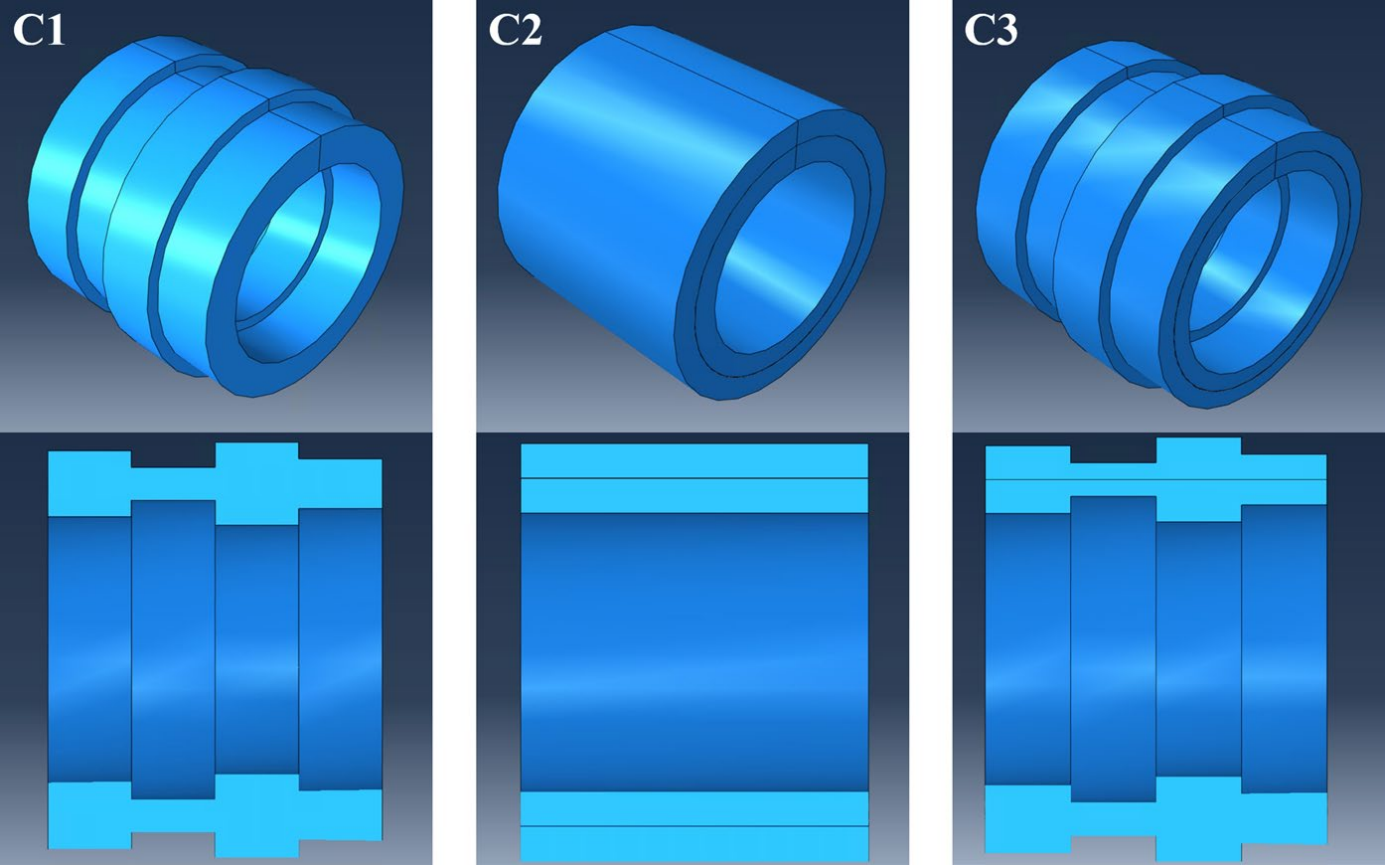
Illustration of cylindrical shells C1, C2 and C3 used for the numerical validation of the model, with their corresponding cross sections

In this section, the predictions made using the methodology outlined above are presented and discussed, with comparisons against numerical and experimental data for validation. Initially, the method was applied to three typical thick circular cylindrical shells, with arbitrary stepped thickness non-uniformities and mechanical properties under free boundary conditions. Detailed description of the dimensions and properties used in these models is presented in Table 2. Cylinder 1, referred to as C1, is an isotropic cylindrical shell with 4 steps of thickness ${h}_{1}, {h}_{2}, {h}_{3}, {h}_{4}$ and corresponding lengths ${L}_{1}, {L}_{2}, {L}_{3}$ and ${L}_{4}$. C2 is a cylinder of constant thickness $h=40 mm$, with an isotropic outer layer ($20 mm$) and an orthotropic inner layer ($20 mm$), and C3 is a combination of C1, with variable thickness and mechanical properties. All Finite Element (FE) models were developed in ABAQUS and are shown in Fig. 5, with their corresponding cross sections.

**Table 2:** Dimensions and mechanical properties of the cylindrical shells used in the preliminary investigation

Parameter		Unit	Cylindrical shell		
			C1	C2	C3
${L}_{1}-{L}_{2}-{L}_{3}-{L}_{4}$		$\mathrm{mm}$	50–50-50–50	50–50-50–50	50–50-50–50
${h}_{1}-{h}_{2}-{h}_{3}-{h}_{4}$		$\mathrm{mm}$	40–20-50–30	40–40-40–40	40–20-50–30
$\mathrm{Radius}$		$\mathrm{mm}$	100	100	100
${E}_{x}$	in	$\mathrm{GPa}$	220	55.6	55.6
	out	$\mathrm{GPa}$	220	69	69
${E}_{\varphi }$	in	$\mathrm{GPa}$	220	198	198
	out	$\mathrm{GPa}$	220	69	69
${E}_{z}$	in	$\mathrm{GPa}$	220	198	198
	out	$\mathrm{GPa}$	220	69	69
$\rho$	in	$\mathrm{kg}/{\mathrm{m}}^{3}$	7850	7600	7600
	out	$\mathrm{kg}/{\mathrm{m}}^{3}$	7850	2700	2700
$\mu$	in	–	0.3	0.3	0.3
	out	–	0.3	0.3	0.3

The modal data obtained for cylinders C1-C3 from the analytical and respective FEA models are presented in Table 3. Upon examining the data, one can see that the average errors for the modes reaching as high as 20 kHz is less than 5%, illustrating the effectiveness and validity of the method. The largest error percentage recorded for each case is 11.81% for mode (0,5) in C1, -11.7% for mode (3,0) in C2, and -8.83% for mode (3,0) in C3. The repetition of mode (3,0) having the highest error in both C2 and C3, as well as both of them having a negative mean error, while C1 has a positive mean error, indicates that possibly the incorporation of the anisotropic material properties introduces a source of error in the analytical model, and may benefit from refinement. However, with a maximum error of 11.81% and mean errors less than 5%, the analytical model performs very well and is deemed suitable for further use and application to the much more sophisticated case of the stator assembly.

**Table 3:** Comparison of natural frequency predictions between the analytical model and FEA for the cylindrical shell case studies used initially

	C1			C2			C3		
Mode $(\mathrm{n},\mathrm{m})$	FEA $(\mathrm{Hz})$	Ana.$(\mathrm{Hz})$	Error $(\mathrm{\%})$	FEA $(\mathrm{Hz})$	Ana.$(\mathrm{Hz})$	Error $(\mathrm{\%})$	FEA $(\mathrm{Hz})$	Ana.$(\mathrm{Hz})$	Error $(\mathrm{\%})$
$(\mathrm{0,0})$	8035	8121	1.07	6771	6978	3.05	6226	6544	5.10
$(\mathrm{0,1})$	8319	8288	− 0.37	8183	8160	− 0.28	7912	8013	1.28
$(\mathrm{0,2})$	8343	8339	− 0.05	8848	8607	− 2.73	8466	8266	− 2.36
$(\mathrm{0,3})$	11,166	12,161	8.91	10,134	10,037	− 0.96	8923	9371	5.02
$(\mathrm{0,4})$	11,892	12,733	7.07	11,611	10,935	− 5.82	10,523	10,125	− 3.78
$(\mathrm{0,5})$	15,058	16,837	11.81	14,130	14,206	0.54	11,226	11,725	4.45
$(\mathrm{1,0})$	5970	6182	3.55	4632	4610	− 0.47	4175	4249	1.77
$(\mathrm{1,1})$	7727	7965	3.08	6620	6625	0.08	6115	6187	1.18
$(\mathrm{1,2})$	10,218	10,511	2.87	8027	8204	2.21	7043	7483	6.25
$(\mathrm{2,0})$	2424	2460	1.49	2422	2211	− 8.71	2306	2151	− 6.72
$(\mathrm{2,1})$	2696	2775	2.93	2731	2551	− 6.59	2426	2311	− 4.74
$(\mathrm{2,2})$	6996	7464	6.69	6232	6157	− 1.20	5124	5242	2.30
$(\mathrm{2,3})$	8776	9178	4.58	6557	6552	− 0.08	6142	6279	2.23
$(\mathrm{3,0})$	6145	6279	2.18	6222	5494	− 11.70	5740	5233	− 8.83
$(\mathrm{3,1})$	6485	6674	2.91	6606	5966	− 9.69	5931	5478	− 7.64
$(\mathrm{3,2})$	9184	9703	5.65	8952	9688	8.22	7767	7329	− 5.64
$(\mathrm{3,3})$	12,003	12,711	5.90	11,652	11,712	0.51	10,574	10,624	0.47
$(\mathrm{4,0})$	10,463	10,763	2.87	9695	9200	− 5.11	9078	8652	− 4.69
$(\mathrm{4,1})$	10,775	11,128	3.28	10,737	9654	− 10.09	9599	8918	− 7.09
$(\mathrm{4,2})$	12,938	13,558	4.79	11,073	11,489	3.76	9936	10,541	6.09
$(\mathrm{4,3})$	15,658	16,565	5.79	13,048	13,045	− 0.02	11,734	12,589	7.29
$(\mathrm{5,0})$	15,053	15,571	3.44	13,038	13,046	0.06	12,442	12,213	− 1.84
$(\mathrm{5,1})$	15,325	15,905	3.78	15,593	15,006	− 3.76	13,483	12,458	− 7.60
$(\mathrm{5,2})$	17,340	18,148	4.66	16,439	16,408	− 0.19	14,066	14,063	− 0.02
$(\mathrm{6,0})$	19,770	20,563	4.01	19,477	18,590	− 4.55	15,741	15,831	0.57
	Mean error $(\%)$		4.12	Mean error $(\%)$		− 2.14	Mean error $(\%)$		− 0.68

Based on this, the convergence of the proposed method is firstly examined by assessing the influence of the connective and boundary virtual spring stiffnesses, the number of Jacobi polynomials, $M$ used in Eq. (45), and the number of shell segments that the assembly is divided into for the natural frequency predictions. Further assessment of the method is performed through comparisons with Experimental Modal Analysis data, as well as numerical results for the free vibration of the assembly under different boundary conditions.

### Convergence studies

**Fig. 6:**
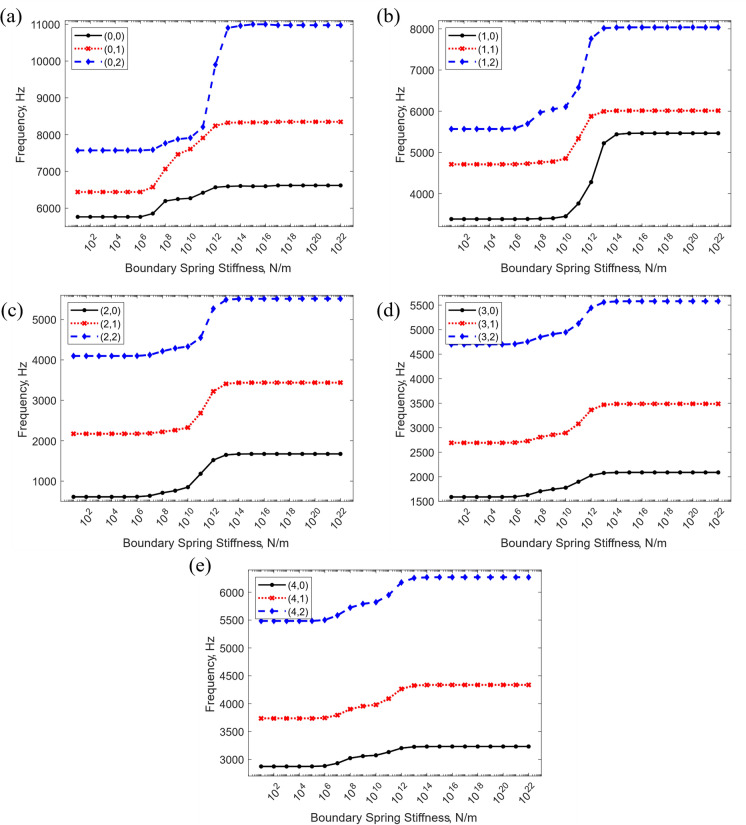
Variation of the stator natural frequencies for different boundary spring stiffness coefficients, for selected vibration mode shapes classified as $(n,m)$, where $n$ denotes the number of circumferential half-waves and $m$ the number of axial half-waves

Fig. 6 shows the effects on the first three vibration modes of the stator when varying the boundary virtual spring stiffness from ${10}^{0}$ to ${10}^{22}\mathrm{N}/\mathrm{m}$ with $n=0$ (Fig. 6a), $n=1$ (Fig. 6b), $n=2$ (Fig. 6c), $n=3$ (Fig. 6d), and $n=4$ (Fig. 6e). The values on the horizontal axis represent the stiffness coefficients of all five springs on both sides of the structure. It can be observed that when ${10}^{0}\mathrm{N}/\text{m }<{k}_{j}<{10}^{6}\mathrm{N}/\mathrm{m}$, the frequencies remain constant, indicating that this region of stiffness coefficient can be used to impose free boundary conditions. For ${k}_{j}>{10}^{14}\mathrm{N}/\mathrm{m}$ the frequencies again remain unchanged and hence any value above this threshold can be used to impose clamping conditions. Within the range ${10}^{7}\mathrm{N}/\text{m }<{k}_{j}<{10}^{13}\mathrm{N}/\mathrm{m}$, the frequencies increase rapidly, suggesting that the virtual springs begin to restrain the motion of the structure. Hence, values within this range can be used to implement elastic constraints at each end of the structure. These findings are also illustrated in [44] and [45]. Another important observation is that in all cases, the higher modes tend to exhibit larger variations with changes in the connective spring stiffness, indicating that they are more susceptible to boundary condition changes. Based on the above, the stiffness values shown in Table 4 were determined for imposing different boundary conditions, to ensure fully converged results.

**Table 4:** Spring stiffness values for different boundary conditions

Boundary condition	Boundary spring stiffness coefficient ($\mathrm{N}/\mathrm{m}$)				
	${k}_{u}$	${k}_{v}$	${k}_{w}$	${k}_{{\beta }_{x}}$	${k}_{{\beta }_{\varphi }}$
Free	$0$	$0$	$0$	$0$	$0$
Simply-supported	${10}^{20}$	${10}^{20}$	${10}^{20}$	$0$	$0$
Clamped	${10}^{20}$	${10}^{20}$	${10}^{20}$	${10}^{20}$	${10}^{20}$

**Fig. 7:**
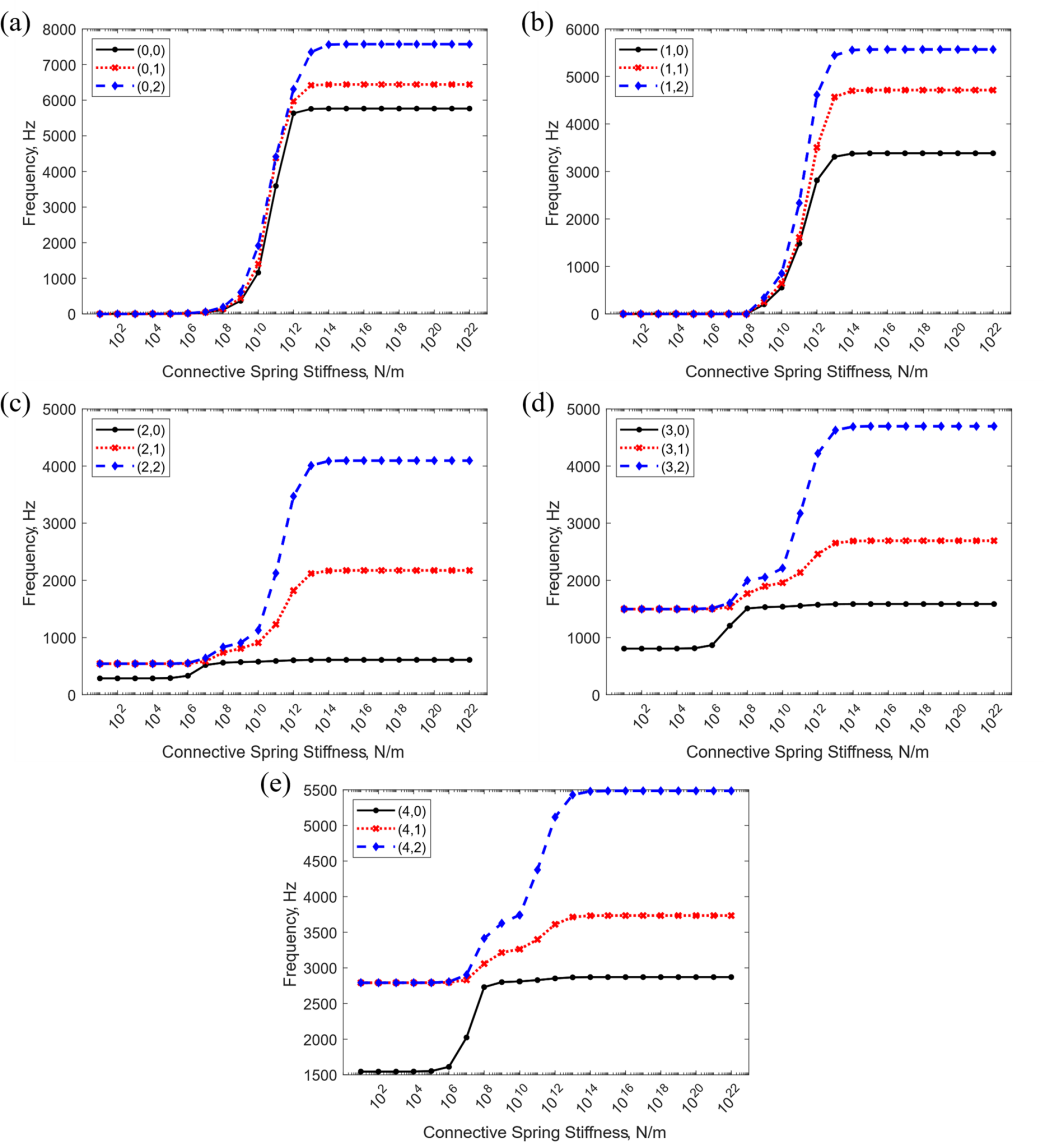
Variation of the stator natural frequencies with different connective spring stiffness coefficients, for selected vibration mode shapes classified as $(n,m)$, where $n$ denotes the number of circumferential half-waves and $m$ the number of axial half-waves

A similar approach was followed to investigate the effect of changing the connective spring stiffness value. The results are illustrated in Fig. 7. Below the threshold stiffness value of ${10}^{6} \mathrm{N}/\mathrm{m}$, the natural frequencies remain relatively low, indicating that the segments are very weakly connected, hence behaving like individual structures. This is further validated when looking at Fig. 7a and b with modes $n=0$ and $n=1$, where below this stiffness value the natural frequency is 0, potentially representing rigid body modes. Similarly, higher vibration modes tend to experience larger fluctuations with stiffness variations. The value of ${10}^{20}\mathrm{N}/\mathrm{m}$ has been selected to impose continuity conditions in the system.

**Fig. 8:**
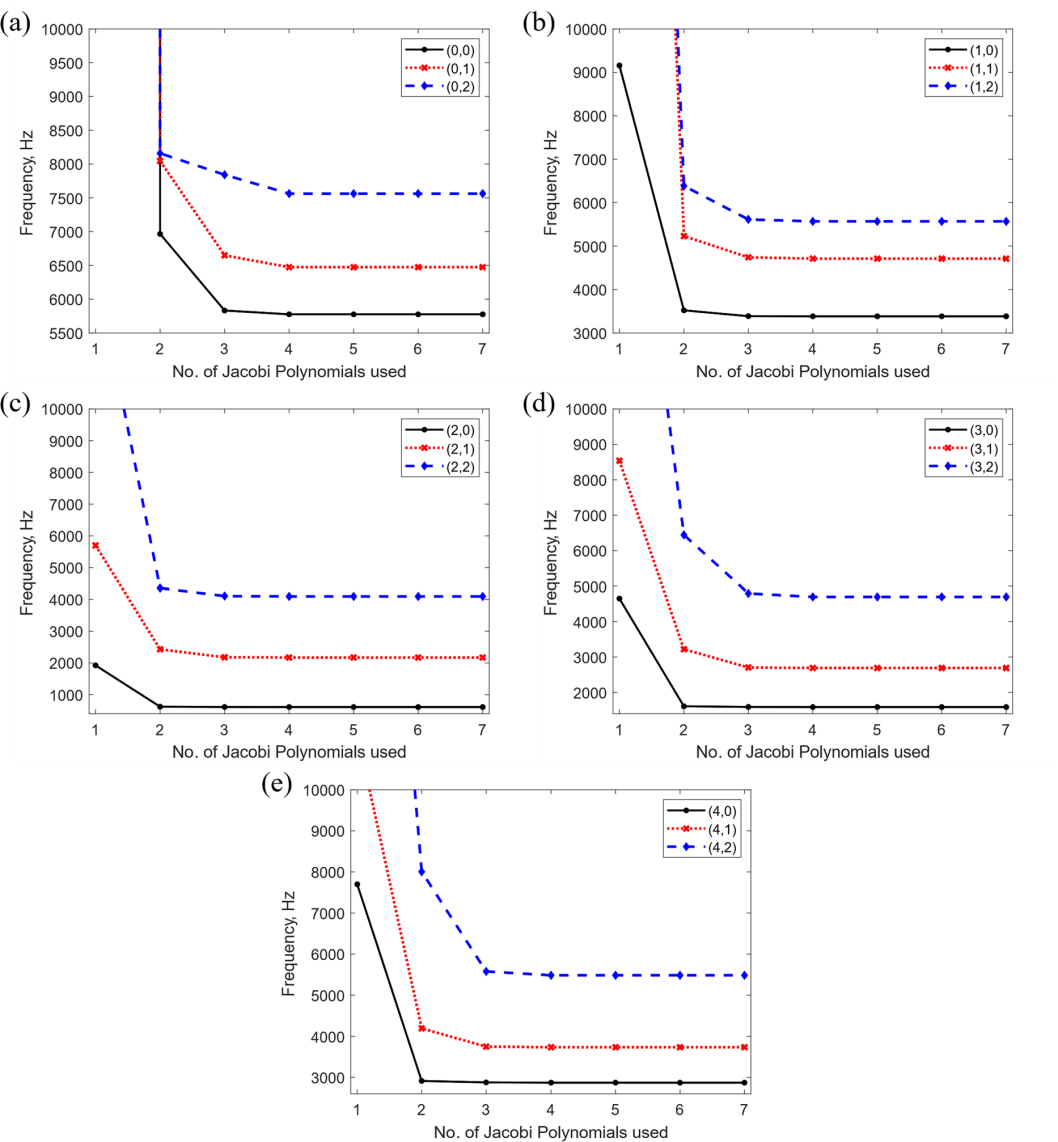
Variation of the stator natural frequencies with different number of Jacobi polynomials, $M$ (Free-Free boundary conditions), for selected vibration mode shapes classified as $(n,m)$, where $n$ denotes the number of circumferential half-waves and $m$ the number of axial half-waves

**Fig. 9:**
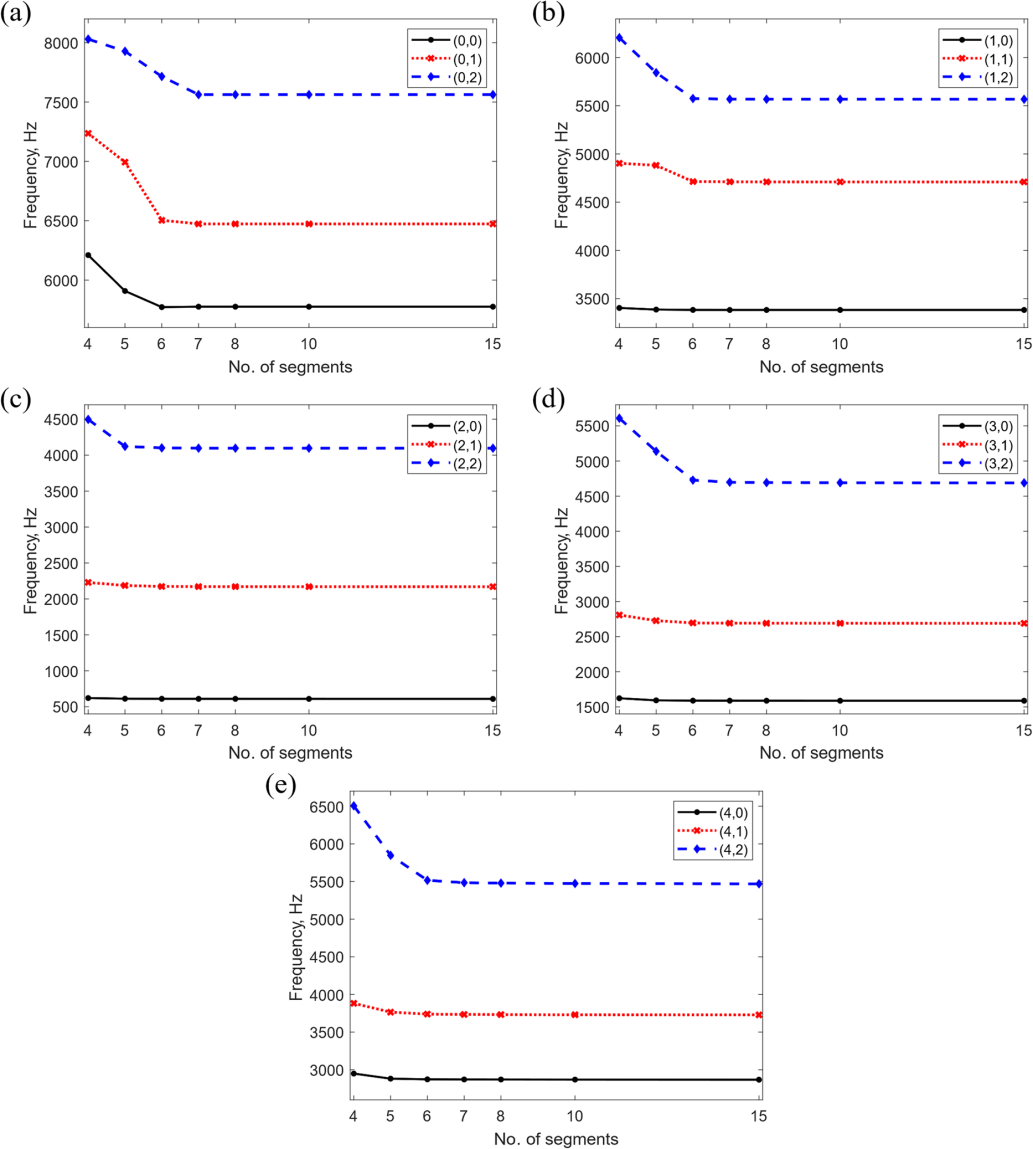
Variation of the stator natural frequencies with different number of shell segments, $P$ (Free-Free boundary conditions), for selected vibration mode shapes classified as $(n,m)$, where $n$ denotes the number of circumferential half-waves and $m$ the number of axial half-waves

Figure 8 illustrates the variation in the natural frequency predictions with respect to the number of truncated Jacobi polynomial terms $M$, used in the admissible functions for free boundary conditions. Similarly, Fig. 9 demonstrates the impact of dividing the stator assembly into varying shell segments $P$, on the free natural frequencies. When $M\ge 3$ and $P\ge 5$, the frequencies are highly stable for all vibration modes and boundary conditions. Hence, to ensure the accuracy of the method yet maintain computational efficiency, the number of shell segments that the stator assembly was divided into, and the number of truncated Jacobi polynomial terms were chosen as $M=4$ and $P=7$. The exact dimensions and mechanical properties used in the representation of this stator assembly are shown in Table 5.

**Table 5:** Properties used in the analytical representation of the stator assembly

Parameter		Unit	Segment						
			1	2	3	4	5	6	7
$\mathrm{Length}, {L}_{p}$		$\mathrm{mm}$	$15$	6	25	45	45	45	25
$\mathrm{Thickness}, {h}_{p}$		$\mathrm{mm}$	26	88	12	14	14	14	6
$\mathrm{Radius}, {R}_{p}$		$\mathrm{mm}$	114	67	104	100	100	100	107
${E}_{x}$	in	$\mathrm{GPa}$	69	69	69	56	56	56	69
	out	$\mathrm{GPa}$	69	69	69	69	69	69	69
${E}_{\varphi }$	in	$\mathrm{GPa}$	69	69	69	198	198	198	69
	out	$\mathrm{GPa}$	69	69	69	69	69	69	69
${E}_{z}$	in	$\mathrm{GPa}$	69	69	69	198	198	198	69
	out	$\mathrm{GPa}$	69	69	69	69	69	69	69
$\rho$	in	$\mathrm{kg}/{\mathrm{m}}^{3}$	5000	2700	2700	16,600	16,600	16,600	2700
	out	$\mathrm{kg}/{\mathrm{m}}^{3}$	2700	2700	2700	2700	2700	2700	2700
$\mu$		–	0.3	0.3	0.3	0.3	0.3	0.3	0.3

### Modal analysis of the stator

**Fig. 10:**
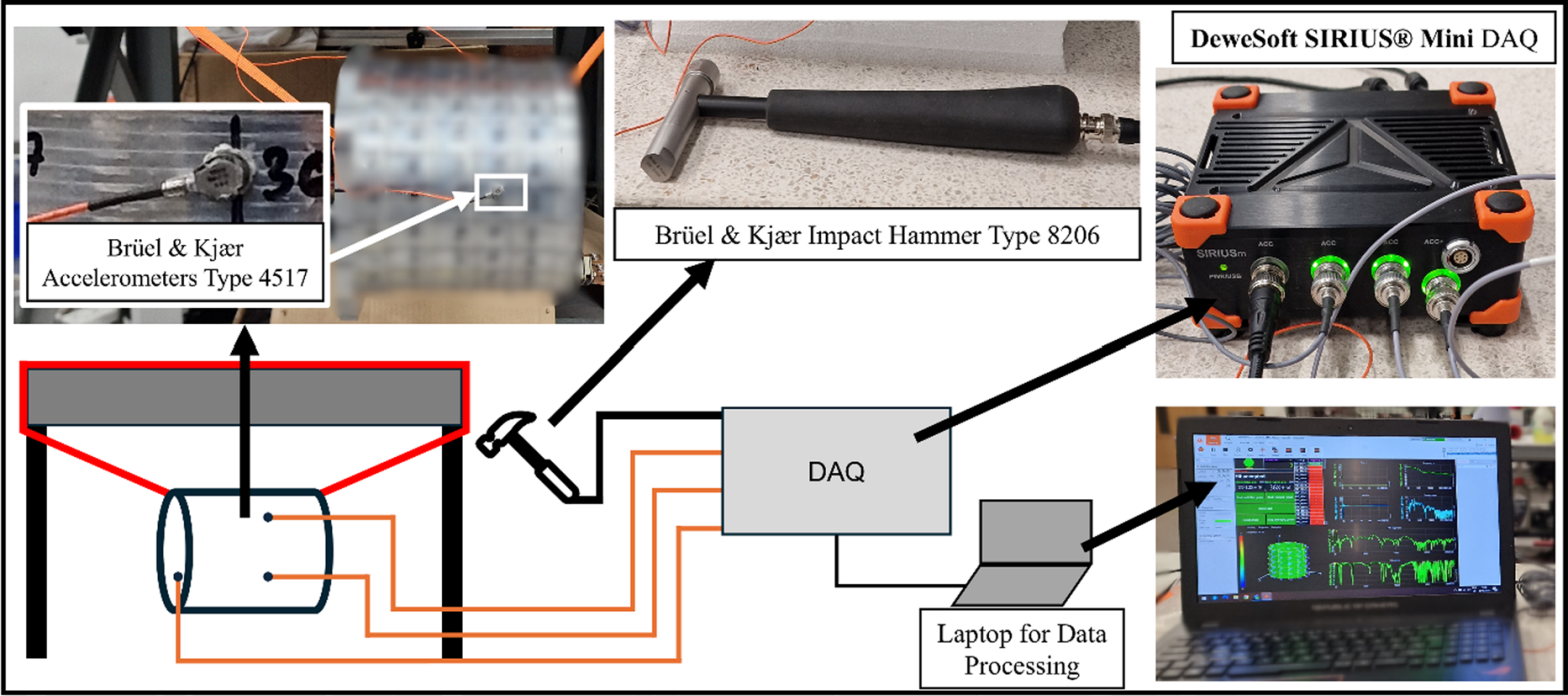
Experimental test setup for modal analysis on stator and cooling jacket assembly using impact hammer (The stator has been blurred to obscure proprietary details of the structure, which were provided under a confidentiality agreement)

To assess the effectiveness of the method in the accurate determination of the natural frequencies of a stator, an experimental modal analysis test rig was set up, as illustrated in Fig. 10. The stator assembly was suspended from a rigid structure using elastic bands to simulate free boundary conditions, and excitation was applied at 192 locations on the outer surface of the cooling jacket, both axially and radially, using an impact hammer. Three uniaxial accelerometers were placed on the structure. The two accelerometers measuring radial accelerations were placed circumferentially at 90° apart, to ensure that circumferential modes were captured correctly. The third accelerometer was placed at the top surface of the cylinder, to measure axial accelerations, ensuring that axial modes were correctly identified.

Data acquisition and post-processing were performed using Dewesoft® hardware and software [61], enabling the extraction of natural frequencies, damping ratios, and mode shapes. Table 6 provides a comparison of the experimentally and analytically determined natural frequencies of the freely suspended system, along with the experimentally computed damping ratios for each corresponding mode. Both the numerical and analytical methods demonstrate a high degree of conformity with the experimentally obtained natural frequencies. Although having a slightly higher mean error relative to FEA, the computational savings made by using the analytical technique, far outweigh the small increase in inaccuracy, as the numerical model required approximately 5 h of computation on a desktop computer with an 8-core processor, while the analytical method produced results within less than 6 min. Reflecting on assumption (iv) in Sect. 2.1, the results of the FEA model of the stator assembly, including the full tooth geometry but utilising only additional mass density to represent the effects of the windings, illustrate that it is a valid assumption that can also be implemented in the analytical model, as the natural frequency predictions are of high accuracy. However, both methods produced a maximum error of 6–8% at specific vibration modes, suggesting that some further mode-specific refinement could still be a possibility to assist the analytical method in achieving even better accuracy.

**Table 6:** Comparison of experimental, FEA and analytical natural frequencies for freely suspended stator assembly

Mode $(n,m)$	Exp.$(\mathrm{Hz})$	Damping $(\mathrm{\%})$	FEA $(\mathrm{Hz})$	FEA error $(\mathrm{\%})$	Ana $(\mathrm{Hz})$	Ana. error $(\%)$
$(\mathrm{2,0})$	604	1.11	647	7.14	609	0.85
$(\mathrm{3,0})$	1692	0.99	1709	1.03	1589	− 6.07
$(\mathrm{2,1})$	2088	1.03	1954	− 6.44	2172	4.00
$(\mathrm{3,1})$	2598	1.05	2515	− 3.20	2692	3.61
$(\mathrm{4,0})$	2976	1.56	3081	3.52	2871	− 3.54
$(\mathrm{1,0})$	3120	0.45	3269	4.77	3383	8.43
$(\mathrm{5,0})$	4721	1.03	4664	− 1.21	4395	− 6.90
$(\mathrm{5,1})$	5472	1.28	5113	− 6.56	5191	− 5.13
$(\mathrm{0,0})$	6004	0.99	5808	− 3.27	5763	− 4.02
$(\mathrm{6,0})$	6387	1.15	6315	− 1.12	6092	− 4.61
$(\mathrm{7,0})$	7991	0.94	7920	− 0.89	7898	− 1.16
$(\mathrm{8,0})$	9316	0.82	9316	0.00	9774	4.92
			Mean Error $(\mathrm{\%})$:	− 0.52	Mean Error $(\mathrm{\%})$:	− 0.80

**Fig. 11:**
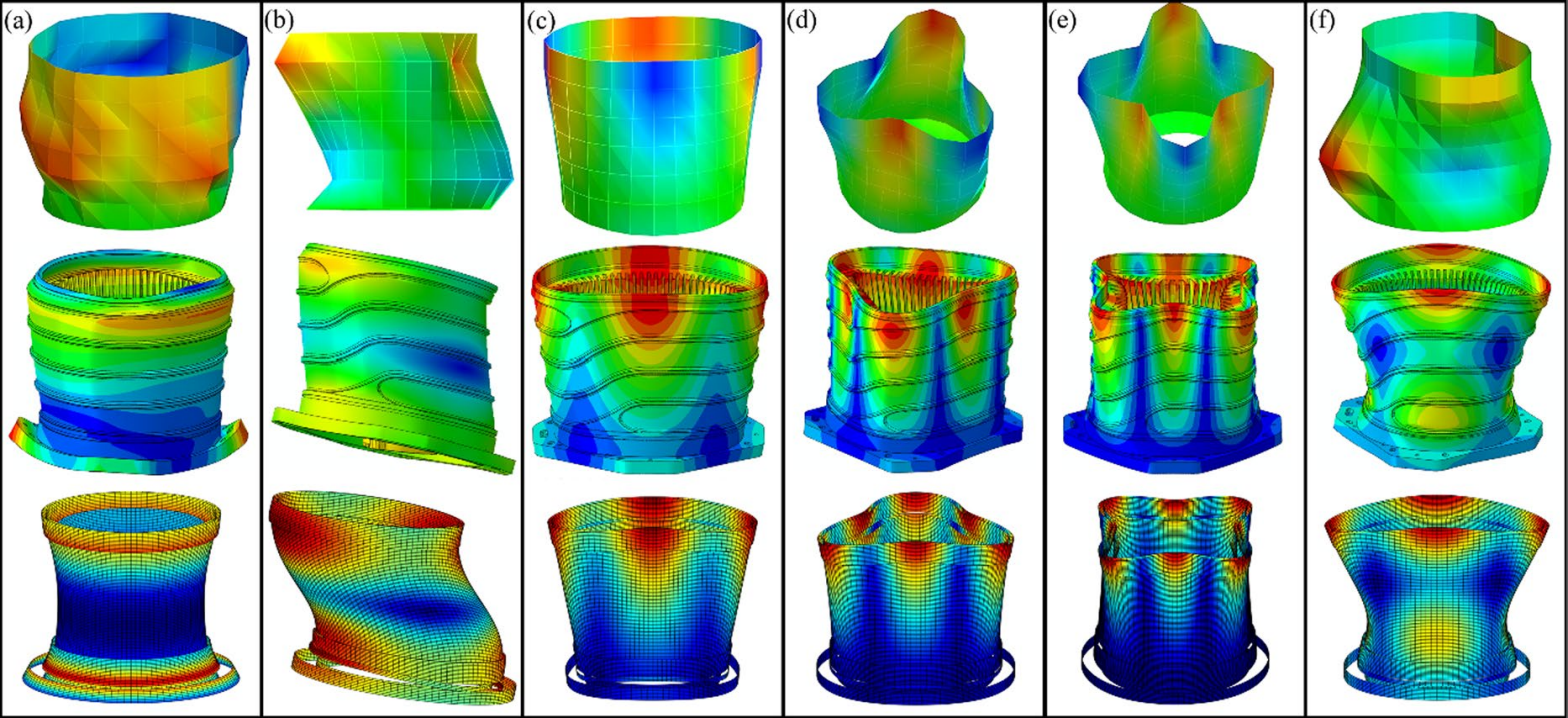
Experimentally (top), numerically (middle) and analytically (bottom) determined mode shapes of the stator assembly, (**a**) mode (0,0), (**b**) mode (1,0), (**c**) mode (2,0), (**d**) mode (3,0), (**e**) mode (4,0) and (**f**) mode (2,1)

In addition to Table 6, Fig. 11 presents a visual comparison of the vibration mode shapes extracted from the experimental measurements (top), FEA analysis (middle) and the proposed method (bottom). The analytical mode shapes were produced by applying the displacement fields on the inner and outer surfaces of the cylindrical shell segments only, hence explaining the appearance of a discontinued system. Nevertheless, the mode shapes show high resemblance to the experimental and numerically produced mode shapes, suggesting that the proposed method is valid and once again provides reassurance on its capabilities.

Having demonstrated the efficacy of the method through the preceding results, the analytical model was used to predict the natural frequencies of the complete stator assembly under more realistic boundary conditions. These include Clamped–Clamped (C–C), Clamped-Free (F-F), Clamped-Simply-supported (C-SS) and Simply-supported-Simply-supported (SS-SS) boundary conditions, which may be more representative of the physical constraints experienced in an EV environment. The results are presented in Table 7. As expected, the frequencies are higher than those in Table 6, due to the additional stiffness introduced to the system through the boundary springs. Once again, the average errors remain below 8%, confirming the method’s robustness. However, a mode-specific evaluation, reveals isolated vibration mode cases where the errors exceed 10%, with a maximum of 30.6%. Closer examination of the results highlights that the highest errors are associated with axisymmetric vibration modes. Upon further inspection of the results, it is clear that the highest errors correspond to mostly axisymmetric modes. This trend of elevated errors in axisymmetric mode predictions has also been observed in previous case studies (cylinders C1, C2, and C3), albeit to a significantly lesser extent. This suggests that part of the observed discrepancy stems from the simplifying assumptions introduced during the development of the methodology. For the axisymmetric case where $n=0$, the assumed displacement field eliminates circumferential shear displacements as $\mathrm{sin}\left(0\varphi \right)=0$, and does not capture shear displacements in the radial-axial plane. In a thick cylindrical shell, however, axisymmetric expansion–contraction modes are accompanied by non-negligible radial shear deformation. The omission of these shear components is therefore expected to contribute to the discrepancies observed in the comparison. For the case of the actual PMSM stator assembly, the increased prediction error is further exacerbated by the inherent axial asymmetry of the structure, which challenges the accuracy of axisymmetric mode identification. It is also worth noting that the non-axisymmetric $(\mathrm{3,2})$ mode exhibits a higher-than-expected deviation. This behaviour can be explained by circumferential non-uniformities in the PMSM stator, such as slotting and asymmetric end features, which can potentially split the degenerate mode pair, as well as by boundary-condition asymmetry at the end fixtures. Furthermore, the $\left(\mathrm{3,2}\right)$ mode has significant shear participation, making it particularly sensitive to the simplified shear properties employed in the model. These combined effects account for the elevated deviation observed in this case, and it is interesting that the influence is most pronounced under clamped–clamped conditions. Again, it may be argued that a refinement of the methodology with targeted modes may assist in improving the accuracy of the method under general boundary conditions. Nonetheless, considering the computational gains from utilising such an approach relatively to numerical simulations, it can be again concluded that the benefits far outweigh the drawbacks.

**Table 7:** Analytically predicted natural frequencies of the stator under different boundary conditions

C − C				C − F			
Mode $(n,m)$	FEA (Hz)	Ana. (Hz)	Error (%)	Mode $(n,m)$	FEA (Hz)	Ana. (Hz)	Error (%)
(2,1)	1384	1446	4.5	(2,0)	902	902	0.0
(3,1)	1918	2089	8.9	(1,0)	1185	1284	8.4
(1,1)	2020	2273	12.5	(3,0)	1746	1627	− 6.8
(2,2)	2837	3129	10.3	(2,1)	2274	2579	13.4
(3,2)	2928	3486	19.1	(3,1)	2593	2758	6.4
(4,1)	3179	3230	1.6	(0,1)	2615	3179	21.6
(0,1)	3743	4887	30.6	(1,1)	2868	3369	17.5
(4,2)	3884	4335	11.6	(4,0)	3090	2878	− 6.9
(1,2)	4099	4390	7.1	(4,1)	3692	3765	2.0
(5,1)	4754	4755	0.0	(1,2)	3877	4130	6.5
(5,2)	5295	5685	7.4	(2,2)	4426	4694	6.1
(3,3)	5344	5578	4.4	(5,0)	4467	4396	− 1.6
(4,3)	5955	6270	5.3	(3,2)	4789	4833	0.9
(1,3)	6223	6013	− 3.4	(5,1)	5128	5204	1.5
(0,2)	6377	6425	0.8	(0,0)	5822	5596	− 3.9
(6,1)	6473	6522	0.8	(1,3)	6031	5602	− 7.1
(6,2)	6870	7344	6.9	(0,1)	6149	5910	− 3.9
(5,3)	6992	7443	6.5	(6,0)	6318	6092	− 3.6
				(6,1)	6687	6899	3.2
				(0,2)	6938	6366	− 8.2
		Mean Error	7.5			Mean Error	2.3

C − SS				SS − SS			
Mode $(n,m)$	FEA (Hz)	Ana. (Hz)	Error (%)	Mode $(n,m)$	FEA (Hz)	Ana. (Hz)	Error (%)
(2,1)	1374	1264	− 8.0	(2,1)	1374	1217	− 11.4
(3,1)	1918	2020	5.3	(3,1)	1918	2011	4.8
(1,1)	2020	2251	11.4	(1,1)	2020	2220	9.9
(2,2)	2837	3007	6.0	(2,2)	2826	2802	− 0.8
(3,2)	2928	3334	13.9	(3,2)	2928	3304	12.8
(4,1)	3174	3163	− 0.3	(4,1)	3174	3160	− 0.4
(0,1)	3743	4847	29.5	(0,1)	3743	4770	27.4
(4,2)	3883	4189	7.9	(4,2)	3883	4177	7.6
(1,2)	4099	4324	5.5	(1,2)	4226	4245	0.4
(5,1)	4754	4690	− 1.3	(5,1)	4754	4690	− 1.3
(5,2)	5295	5555	4.9	(5,2)	5295	5554	4.9
(3,3)	5352	5380	0.5	(3,3)	5340	5315	− 0.5
(4,3)	5955	6085	2.2	(4,3)	5955	6059	1.7
(1,3)	6223	5844	− 6.1	(1,3)	6223	5650	− 9.2
(0,1)	6377	6367	− 0.2	(0,1)	6377	6226	− 2.4
(6,1)	6470	6460	− 0.2	(6,1)	6470	6460	− 0.2
(6,2)	6870	7236	5.3	(6,2)	6870	7233	5.3
(5,3)	6992	7283	4.2	(5,3)	6992	7273	4.0
		Mean Error	4.5			Mean Error	2.9

## Conclusions

Addressing the limitations of existing analytical and numerical modelling techniques, this study introduces a robust and precise eigenvalue analysis framework, considering the stator assembly as a thick cylindrical shell with material anisotropies and dimensional non-uniformities. The method is applied to various structures, and the calculations are extensively validated with both numerical and experimental means. The following conclusions are drawn:

1. By employing First-Order Shear Deformation Shell Theory (FSDST), including shear deformation, rotary inertia, and a trapezoidal stress distribution, the proposed method accurately captures a wide range of vibration modes, including axial, circumferential, torsional, and bending. Combined with the well-established Jacobi-Ritz solution approach, this yields high accuracy and over 95% reduction in computational time relative to FEA, making it especially suitable for rapid early-stage design and optimisation.
2. A significant novelty of the proposed approach lies in its ability to predict axisymmetric (breathing) modes in thick, non-uniform cylindrical shells with arbitrary boundary conditions—an area not previously addressed with comparable generality in the literature. These modes are critical for NVH analysis of electric motors, and the close agreement with FEA and EMA mode shapes highlights the method’s effectiveness.
3. The approach incorporates both continuous and stepped property variations through continuous functions, allowing for detailed representation of material and geometric transitions. The treatment of teeth and windings as equivalent added masses has been validated through strong frequency and mode shape agreement.
4. Axial segmentation of the assembly and utilisation of artificial connective springs allows for efficient modelling of axially stepped thickness variations and fully arbitrary boundary conditions, as evidenced by the strong agreement between the analytical results and the numerical and experimental data.
5. The numerical and experimental data presented demonstrate strong agreement with the analytical predictions, with typical deviations remaining below 5%. While further refinements targeting specific modes could enhance accuracy in cases where discrepancies are higher, the method remains highly promising as a powerful tool for NVH engineers.

Overall, this study provides a versatile, efficient, and accurate analytical framework for eigenvalue analysis of complex cylindrical structures, offering significant potential for advancing the understanding and design of systems in engineering applications. Further advancements could include incorporating forced vibration analyses under steady-state or transient electromagnetic excitations to establish a comprehensive NVH modelling approach for e-motors. Moreover, integrating multi-physics coupling effects, such as thermo-mechanical interactions, would significantly enhance its applicability to cutting-edge motor designs, bridging the gap between analytical precision and real-world behaviour.

## Conflict of interest

The authors declare no competing interests.

## Data Availability

No datasets were generated or analysed during the current study.

## Abbreviations

ANA: Analytical
EMA: Experimental modal analysis
EV: Electric vehicle
FEA: Finite element analysis
FGM: Functionally graded material
FSDST: First order shear deformation stress theory
HSDST: Higher-order shear deformation stress theory
NVH: Noise, vibration & harshness
PMSM: Permanent magnet synchronous machine

## Symbols

${{B}_{x}}_{mn}^{p}$: Generalised coordinate of shear axial displacement of shell segment $p$ for vibration mode $(m,n)$
${{\rm B}_{\varphi }}_{mn}^{p}$: Generalised coordinate of shear tangential displacement of shell segment $p$ for vibration mode $(m,n)$
${E}_{l}$: Elastic modulus of lamination material, $\mathrm{Pa}$
${E}_{s}$: Elastic modulus of silicon steel, $\mathrm{Pa}$
${E}_{x}$: Equivalent elastic modulus in the axial direction, $\mathrm{Pa}$
${E}_{z}$: Equivalent elastic modulus in radial direction, $\mathrm{Pa}$
${E}_{\varphi }$: Equivalent elastic modulus in tangential direction, $\mathrm{Pa}$
${\boldsymbol{F}}$: External excitation vector
${f}^{p}$: External load acting on ${p}^{th}$ shell segment, $\mathrm{N}$
${G}_{xz}$: Equivalent shear modulus in the axial-tangential plane, $\mathrm{Pa}$
${G}_{x\varphi }$: Equivalent shear modulus in the axial-tangential plane, $\mathrm{Pa}$
${G}_{z\varphi }$: Equivalent shear modulus in the radial-tangential plane, $\mathrm{Pa}$
${h}_{p}$: Thickness of ${p}^{th}$ segment, $\mathrm{m}$
${k}_{u,v,\dots }$: Artificial massless spring constant for corresponding displacement component, $\mathrm{N}/\mathrm{m}$
${\boldsymbol{K}}$: Generalised stiffness matrix
$L$: Shell total length, $\mathrm{m}$
$\mathcal{L}$: Lagrangian Energy function, $\mathrm{J}$
${L}_{p}$: Length of ${p}^{th}$ shell segment, $\mathrm{m}$
$m$: Axial wave number of cylindrical mode shape
${m}_{w}$: Mass of windings, $\mathrm{kg}$
${\boldsymbol{M}}$: Generalised mass matrix
$n$: Circumferential wave number of cylindrical mode shape
$$O$$: Origin
$P$: Number of shell segments, $\mathrm{m}$
${P}_{m}^{\left(\alpha ,\beta \right)}$: Jacobi polynomial function of the ${m}^{th}$ order
${\boldsymbol{q}}$: Global generalised coordinate vector
$\ddot{{\boldsymbol{q}}}$: Second time derivative of generalised coordinate vector
${Q}_{ij}^{p}$: Elastic stiffness coefficient of ${p}^{th}$ shell segment
$$R$$: Shell radius, $$\mathrm{m}$$
$$t$$: Time, $$\mathrm{s}$$
${T}^{p}$: Kinetic energy of ${p}^{th}$ shell segment, $\mathrm{J}$
${u}_{0}^{p}$: Transverse axial mid-surface displacement of ${p}^{th}$ shell segment, $\mathrm{m}$
${u}^{p}$: Axial displacement at given coordinates of ${p}^{th}$ shell segment, $\mathrm{m}$
${U}_{b}$: Boundary energy from artificial massless springs, $\mathrm{J}$
${U}_{c}^{p,p+1}$: Connective spring energy between shell segments $p$ and $p+1$, $\mathrm{J}$
${U}_{mn}^{p}$: Generalised coordinate of transverse axial displacement of shell segment $p$ for vibration mode $(m,n)$
${U}^{p}$: Elastic strain energy of ${p}^{th}$ shell segment, $\mathrm{J}$
${v}_{0}^{p}$: Transverse tangential mid-surface displacement of ${p}^{th}$ shell segment, $\mathrm{m}$
${v}^{p}$: Tangential displacement at given coordinates of ${p}^{th}$ shell segment, $\mathrm{m}$
${V}_{l}$: Volume of lamination material, ${\mathrm{m}}^{3}$
${V}_{mn}^{p}$: Generalised coordinate of transverse tangential displacement of shell segment $p$ for vibration mode $(m,n)$
${V}_{s}$: Volume of silicon steel, ${\mathrm{m}}^{3}$
${V}_{\mathrm{teeth}}$: Volume of stator teeth, ${\mathrm{m}}^{3}$
${w}_{0}^{p}$: Transverse radial mid-surface displacement of ${p}^{th}$ shell segment, $\mathrm{m}$
${w}^{p}$: Radial displacement at given coordinates of ${p}^{th}$ shell segment, $\mathrm{m}$
${W}_{mn}^{p}$: Generalised coordinate of transverse radial displacement of shell segment $p$ for vibration mode $(m,n)$
${W}^{p}$: External work done on ${p}^{th}$ shell segment, $\mathrm{J}$
$x$: Axial coordinate system direction, $\mathrm{m}$
$z$: Radial (normal) coordinate system direction, $\mathrm{m}$

## Greek letters

$\alpha ,\beta$: Jacobi polynomial coefficients
${\beta }_{x}^{p}$: Shear mid-surface displacement in axial direction of ${p}^{th}$ shell segment, $\mathrm{m}$
${\beta }_{\varphi }^{p}$: Shear mid-surface displacement in tangential direction of ${p}^{th}$ shell segment, $\mathrm{m}$
$\gamma$: Shear strain component
${\gamma }^{0}$: Mid-surface shear strain component
$\varepsilon$: Normal strain component
${\varepsilon }^{0}$: Mid-surface normal strain component
$\mathcal{H}\left(\right)$: Heaviside Theta function
$\kappa$: Mid-surface curvature
$$\mu$$: Poisson’s ratio
$$\rho$$: Density, $$\mathrm{kg}/{\mathrm{m}}^{3}$$
$\sigma$: Normal stress component, $\mathrm{Pa}$
$\tau$: Shear stress component, $\mathrm{Pa}$
$\varphi$: Tangential coordinate system direction, $\mathrm{m}$
$$\omega$$: Angular frequency, $$rad/s$$
${\omega }_{mn}$: Natural frequency of vibration mode $(m,n)$, $rad/s$
